# Radiofrequency and Microwave Ablation Compared to Systemic Chemotherapy and to Partial Hepatectomy in the Treatment of Colorectal Liver Metastases: A Systematic Review and Meta-Analysis

**DOI:** 10.1007/s00270-018-1959-3

**Published:** 2018-04-17

**Authors:** Martijn R. Meijerink, Robbert S. Puijk, Aukje A. J. M. van Tilborg, Kirsten Holdt Henningsen, Llenalia Garcia Fernandez, Mattias Neyt, Juanita Heymans, Jacqueline S. Frankema, Koert P. de Jong, Dick J. Richel, Warner Prevoo, Joan Vlayen

**Affiliations:** 1 Department of Radiology, VU University Medical Centre, De Boelelaan 1117, 1081 HV Amsterdam, The Netherlands; 2 Medical Evaluation and Technology Assessment (ME-TA), Rotselaar, Belgium; 3 National Health Care Institute Netherlands (ZiNL), Diemen, The Netherlands; 4 Department of Surgery, University Medical Center Groningen, Groningen, The Netherlands; 5 Department of Oncology, Medisch Spectrum Twente, Enschede, The Netherlands; 6 Department of Radiology, Antoni van Leeuwenhoek Hospital, Amsterdam, The Netherlands

**Keywords:** Colorectal liver metastases (CRLM), Radiofrequency ablation (RFA), Microwave ablation (MWA), Partial hepatectomy (PH), Systemic chemotherapy

## Abstract

**Purpose:**

To assess safety and outcome of radiofrequency ablation (RFA) and microwave ablation (MWA) as compared to systemic chemotherapy and partial hepatectomy (PH) in the treatment of colorectal liver metastases (CRLM).

**Methods:**

MEDLINE, Embase and the Cochrane Library were searched. Randomized trials and comparative observational studies with multivariate analysis and/or matching were included. Guidelines from National Guideline Clearinghouse and Guidelines International Network were assessed using the AGREE II instrument.

**Results:**

The search revealed 3530 records; 328 were selected for full-text review; 48 were included: 8 systematic reviews, 2 randomized studies, 26 comparative observational studies, 2 guideline-articles and 10 case series; in addition 13 guidelines were evaluated. Literature to assess the effectiveness of ablation was limited. RFA + systemic chemotherapy was superior to chemotherapy alone. PH was superior to RFA alone but not to RFA + PH or to MWA. Compared to PH, RFA showed fewer complications, MWA did not. Outcomes were subject to residual confounding since ablation was only employed for unresectable disease.

**Conclusion:**

The results from the EORTC-CLOCC trial, the comparable survival for ablation + PH versus PH alone, the potential to induce long-term disease control and the low complication rate argue in favour of ablation over chemotherapy alone. Further randomized comparisons of ablation to current-day chemotherapy alone should therefore be considered unethical. Hence, the highest achievable level of evidence for unresectable CRLM seems reached. The apparent selection bias from previous studies and the superior safety profile mandate the setup of randomized controlled trials comparing ablation to surgery.

**Electronic supplementary material:**

The online version of this article (10.1007/s00270-018-1959-3) contains supplementary material, which is available to authorized users.

## Introduction

Colorectal cancer is the second most common cause of cancer-related death in developed countries and the third most common malignancy worldwide [1]. Roughly 50% of patients develop colorectal liver metastases (CRLM), yet only a minority (10–15%) can undergo partial hepatectomy (PH). Five-year survival following PH ranges between 31 and 58% in carefully selected patients [2, 3]. The remainder is usually offered chemotherapy and/or local tumour ablation alone or in combination with PH. Especially radiofrequency (RFA) and microwave ablation (MWA) are commonly employed and widely available. Median overall survival (OS) following systemic treatment nowadays reaches 20–22 months in patients who receive sequential chemotherapy regimens often with biological agents; 5-year survival remains < 15% [4–8]. Five-year survival following ablation varies between 17 and 53% [9–13]. Although recent studies [13–16] have reported similar survival for patients treated with thermal ablation or PH, interventional radiology and surgical oncology communities generally state that thermal ablation cannot be considered an alternative to PH. They recommend the use of open, laparoscopic or percutaneous RFA and MWA for small CRLM (≤ 3 cm) in patients who are unsuitable for resection due to (1) an impaired general health status (age, comorbidities), (2) a history of extensive abdominal surgery, (3) the presence of lesions with an unfavourable location or (4) an insufficient future liver remnant to resect all lesions [11, 17, 18]. In light of these recommendations the Dutch National Health Care Institute (ZiNL) and representatives from the Dutch societies for interventional radiology, surgical and medical oncology commissioned a systematic review and meta-analysis with the following research questions: (1) what is the evidence regarding safety and effectiveness for RFA and MWA in the treatment of CRLM? and (2) what is the status of RFA and MWA in international guidelines?

## Materials and Methods

### Search Strategies

The search strategies and inclusion criteria were based on the following PICOS question: P (population): patients with resectable and unresectable CRLM; I (intervention): RFA and MWA; C (comparison): for resectable disease PH and for unresectable disease systemic chemotherapy; O (outcomes): critical endpoints were OS, complications and quality of life (QoL), important endpoints were disease-free survival (DFS), local progression-free survival (LPFS), and ablation-site recurrence rate (ASR); S (study designs): (systematic reviews), randomized studies, controlled studies, comparative observational studies with multivariate analysis and/or matching, non-comparative studies if an insufficient number of comparative studies was found. To assess the relative importance of outcomes (critical, important but not critical or limited) the Grading of Recommendations, Assessment, Development and Evaluation (GRADE) approach was used [19].

We used Cochrane systematic review methods to identify studies that met the inclusion criteria. MEDLINE, Embase and the Cochrane Library (Cochrane Database of Systematic Reviews, Database of Abstracts of Reviews of Effectiveness, Health Technology Assessment database, CENTRAL) were searched (last update September 26th 2017) using a combination of text words and medical subheadings (search strategies: Table 3 online appendix*)*. No time limit was used.

Searches were limited to studies involving humans and published in English or Dutch. Abstracts were only taken into consideration when their methodological quality could be sufficiently evaluated and data extraction could be entirely completed. Studies also describing primary liver tumours and/or non-colorectal liver metastases were only included if data about CRLM could be extracted separately. Only studies reporting on the following outcomes were considered: (1) critical outcomes: OS, QoL and complications; (2) important outcomes: DFS, LPFS, ASR.

### Study Selection and Quality Criteria

All retrieved studies were evaluated for inclusion by two reviewers (JV, KHH) independently. First, studies were evaluated on title and abstract. Studies potentially eligible for inclusion were ordered in full text for a comprehensive evaluation.

For the included studies, the methodological quality was evaluated independently using the AMSTAR tool for systematic reviews and the risk of bias tool of the Cochrane Collaboration for randomized trials and controlled studies. For uncontrolled studies (including case series) the following criteria were judged: adequate definition of disease, clear baseline characteristics, inclusion of a representative cohort, adequate disease confirmation using validated methods, standardized data collection and objective outcome measurement.

All discrepancies were resolved by consensus. If no consensus was reached, the opinion of a third researcher (LGF) was the overriding factor.

### Data Extraction

Data were extracted by one reviewer (KHH or LGF) and checked by a second (JV). The results were displayed as described in the article, allowing for recalculations based on the data extracted from the article if needed.

### Data Analysis

Based on clinical criteria, such as population, intervention, control group and outcome, an assessment was made whether the studies were sufficiently comparable to perform a meta-analysis. A random effects model was chosen, unless there was no statistical heterogeneity. Individual results were presented in a forest plot. The following comparisons and outcomes allowed for a meta-analysis: (1) RFA versus PH alone regarding OS, DFS, LPFS, 30-day mortality and complications, and (2) RFA + PH versus PH alone regarding OS, DFS, LPFS and 60-day mortality. For time-to-event outcomes (survival), the generic inverse variance method was used. Only corrected hazard ratios (HR; e.g. based on a multivariate analysis) were imputed. For dichotomic results (complications), the Mantel–Haenszel method was used to calculate risk ratios (RR). When ≥ 10 studies were available for inclusion in the meta-analysis a funnel plot was used to assess for publication bias. The meta-analysis was conducted using Review Manager 5.3.

### Levels of Evidence

To appoint a level of evidence, the GRADE system was used taking into account the quality assessment and the results from data extraction [20, 21]. We classified the level of evidence into 4 GRADE categories: high, moderate, low and very low (Table 1). Quality elements evaluated for downgrading were study limitations, inconsistency, indirectness, imprecision and publication bias.

**Table 1:** Grading of Recommendations Assessment, Development and Evaluation (GRADE^*^) [19, 20]

Endpoint	Conclusion	Literature review	GRADE level
Overall survival	RFA (± PH) + chemotherapy is superior to chemotherapy alone	1 RCT (downgraded; serious imprecision)^a^	Moderate
	RFA + chemotherapy is superior to chemotherapy alone	1 RCT (downgraded 2*x*; serious indirectness^b^ and serious imprecision)^a^	Low
	RFA (for unresectable CRLM) + PH is equivalent to PH alone	Observational comparative studies	Very low
	RFA alone (for unresectable CRLM) is inferior to PH alone	Observational comparative studies	Very low
	MWA is equivalent to PH	1 RCT (downgraded; very serious risk of bias)	Very low
	MWA (for unresectable CRLM) + PH is equivalent to PH alone	One observational comparative study	Very low
Complications	RFA alone (for unresectable CRLM) is superior to PH	Observational comparative studies	Very low
	Studies on RFA (for unresectable CRLM) + PH versus PH alone show conflicting results	Observational comparative studies	–
	MWA alone is equivalent to PH	1 RCT (downgraded; very serious risk of bias)	Very low
Quality of life	There are no comparative studies on the effect of RFA or MWA	–	–

^*^GRADE definitions: *high quality*—further research is very unlikely to change our confidence in the estimate of effect (randomized controlled trials); *moderate quality*—further research is likely to have an important impact on our confidence in the estimate of effect and may change the estimate (controlled trials, no randomization), *low quality*—further research is very likely to have an important impact on our confidence in the estimate of effect and is likely to change the estimate (observational studies); *very low quality*—any estimate of effect is very uncertain (any other type)

^a^serious imprecision: in case of low optimal information size (OIS; number of included patients did not meet sample size), dichotomous outcomes, low number of events, wide confidence intervals with uncertainty about magnitude of effect, or when there is a lot of variation in the effects among the participants in continious measures

^b^serious indirectness: very important differences in populations, interventions, outcome measures, or indirect comparisons

Two independent researchers graded the evidence levels (JV, KHH). If consensus was not reached, the opinion of a third independent researcher was decisive (LGF). The reasons for appointing evidence levels were documented.

### Guidelines

(Inter)national guidelines about RFA and MWA for CRLM were searched in the following database: National Guideline Clearinghouse and Guidelines International Network as well as on websites of (inter)national guideline organizations and scientific societies. Two reviewers (JV, LGF) selected and judged the guidelines using the AGREE II instrument (Table 2 online appendix) [22]. If consensus was not reached, the opinion of a third independent researcher (KHH) was decisive.

## Results

**Fig. 1:**
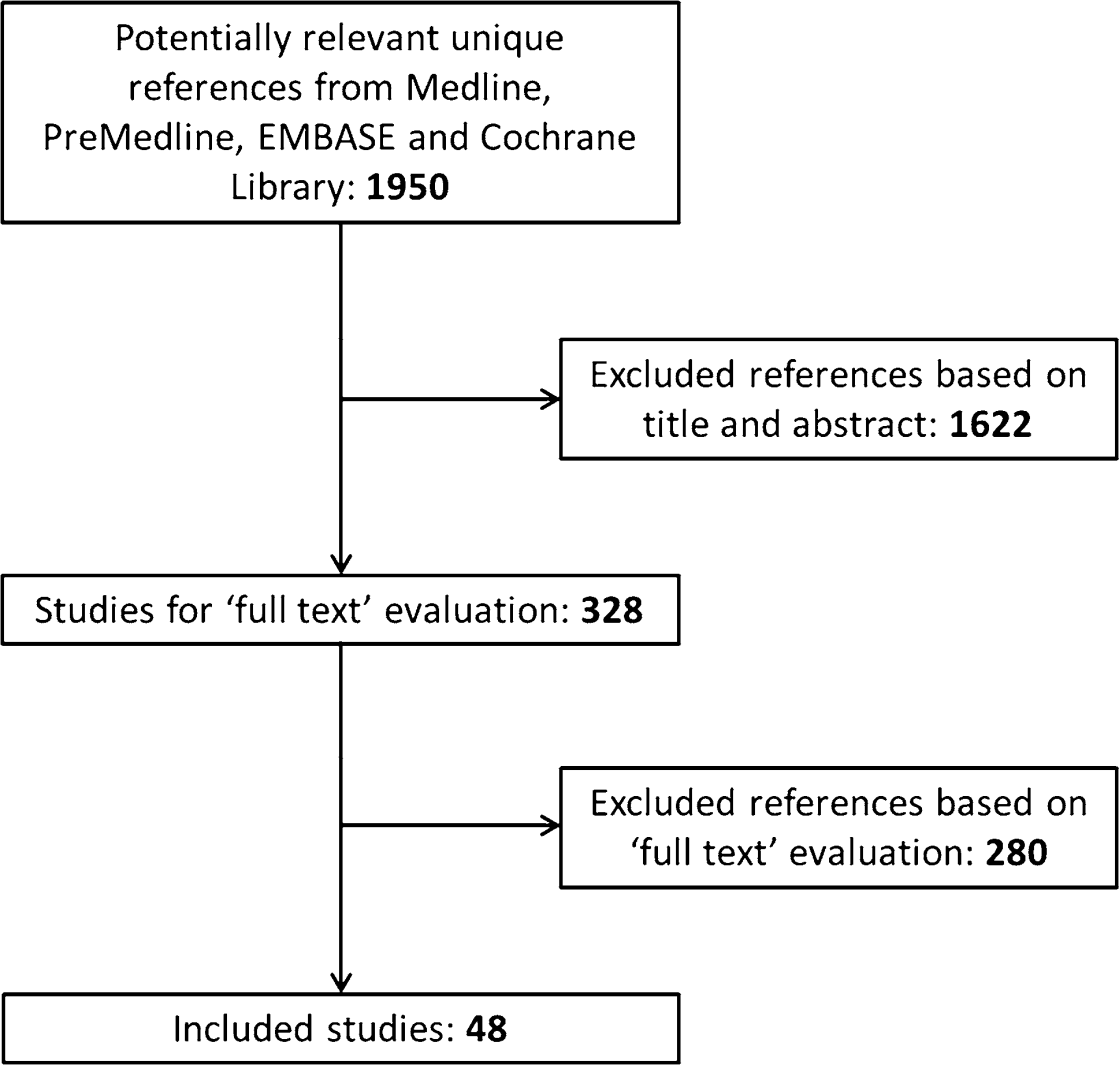
Results of selection: effectiveness of thermal ablation versus surgical resection or systemic chemotherapy in treating patients with CRLM

**Fig. 2:**
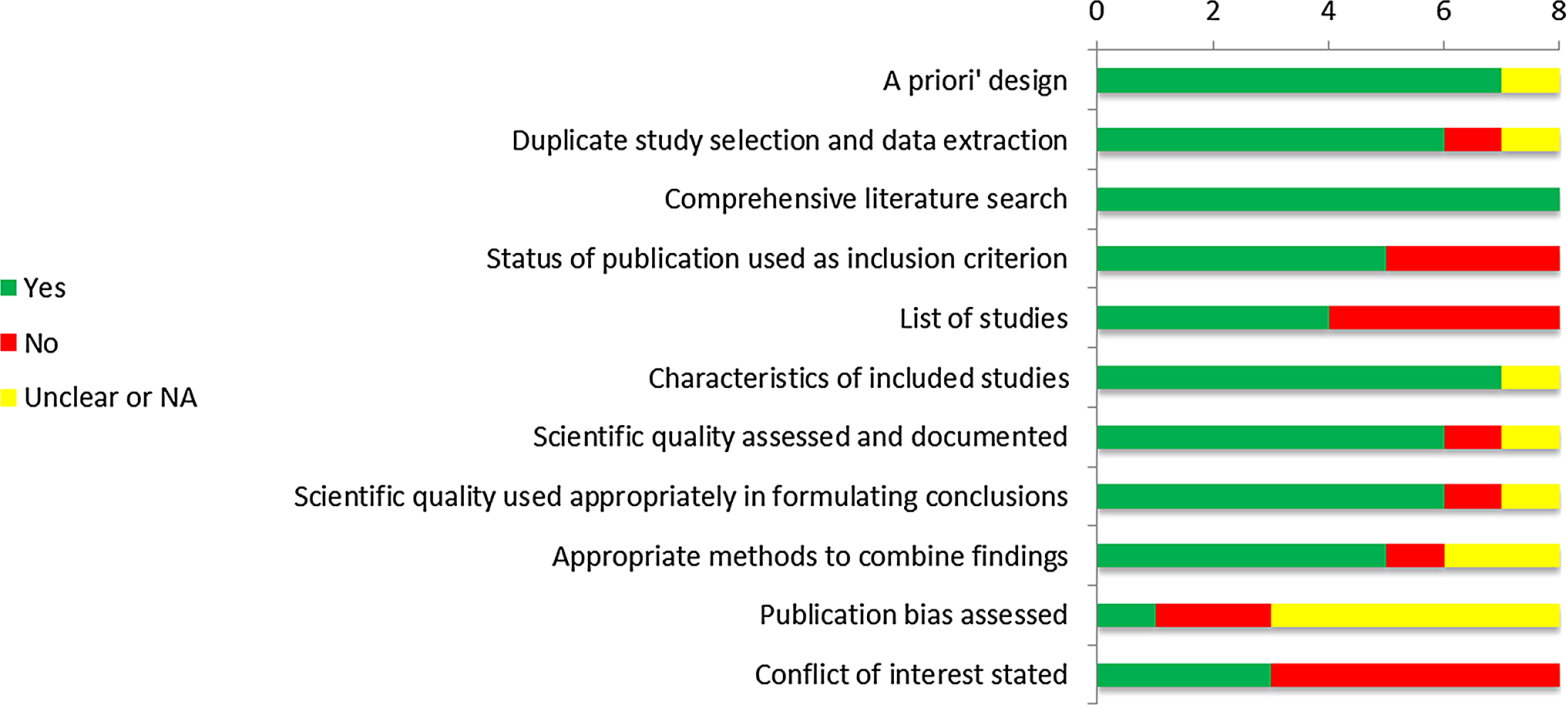
Risk of bias of included reviews for RFA

The literature search resulted in 3530 records. After excluding 1121 duplicate papers and 459 documents written in a non-English language, a total of 1950 unique references remained (Fig. 1). Based on title and abstract 1622 references were excluded. A total of 328 articles were selected for full-text review. This led to the exclusion of 280 articles for the following reasons: single cohort without comparison (*n* = 115); wrong comparator, comparison, intervention or outcome (*n* = 48); no separate results for CRLM (*n* = 22); systematic review without quality appraisal (*n* = 20); narrative review (*n* = 17); observational study without matching or multivariate analysis (*n* = 16); and other (*n* = 42) (Table 4 online appendix). A total of 48 articles were included: eight systematic reviews, two randomized studies, twenty-six comparative observational studies and ten case series. Two references were included as guideline. Seven out of eight systematic reviews were classified as high quality [1–3, 9, 23–25], one was judged as poor quality [26] (Fig. 2).

Updated search resulted in three new comparative observational studies [13, 27, 28].

### RFA

**Fig. 3:**
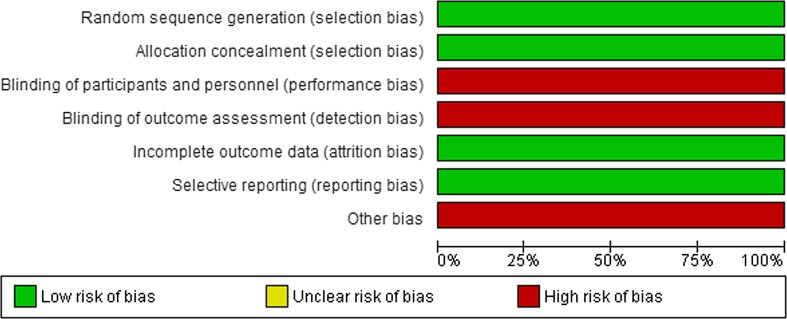
Risk of bias of Ruers et al. [29]

One randomized controlled trial (EORTC-CLOCC trial) compared systemic chemotherapy (FOLFOX [Folinic acid, Fluorouracil, Oxaliplatin] and from October 2005 FOLFOX + bevacizumab) with or without RFA in 119 patients with unresectable CRLM (Fig. 3) [29]. Median number of CRLM was 4 (systemic + RFA) and 5 (systemic alone); 25.0% of patients in systemic + RFA group had solitary metastases, 11.9% in the systemic only group. Due to slow recruitment the trial was downgraded to a phase II study.

**Fig. 4:**
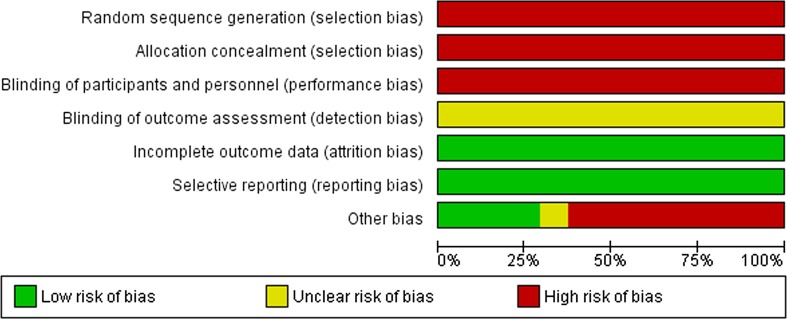
Risk of bias of comparative observational studies for RFA

Twenty-four observational studies compared RFA for unresectable CRLM to PH for resectable disease (Fig. 4). Fourteen studies compared RFA with surgery alone [13, 30–42], eight studies compared RFA + PH with PH alone [13, 15, 16, 18, 27, 28, 43, 44], and four studies compared RFA to RFA + PH or PH alone [13, 45–47]. A total number of 5020 patients were included in these observational studies (RFA: *N* = 1103; RFA + PH: *N* = 541; PH alone: *N* = 3376). For none of these studies, it could be excluded that therapy selection was based on patient and/or tumour characteristics and/or physician preference (confounding by indication). Moreover, the methods used to describe outcomes were heterogeneous and, although all included studies used multivariate analysis or data matching based on prognostic factors, these factors differed from study to study. None of the studies blinded patients or outcome assessors. In eleven studies, data collection was retrospective.

### Overall Survival

#### RFA Plus Chemotherapy Versus Chemotherapy Alone

The EORTC-CLOCC trial reported a 30-month OS of 61.7% (95% confidence interval (CI) 48.2–73.9%) for the combination group versus 57.6% (95%CI 44.1–70.4%) in the chemotherapy alone group [29]. After a median follow-up of 9.7 years, OS was significantly better in the RFA + chemotherapy group (HR = 0.58; 95%CI 0.38–0.88) with an 8-year OS of 35.9 versus 8.9% for chemotherapy alone [29]. In the RFA arm 27 out of 50 patients also underwent hepatic resection(s) which may have confounded results.

#### RFA Versus PH Alone

**Fig. 5:**
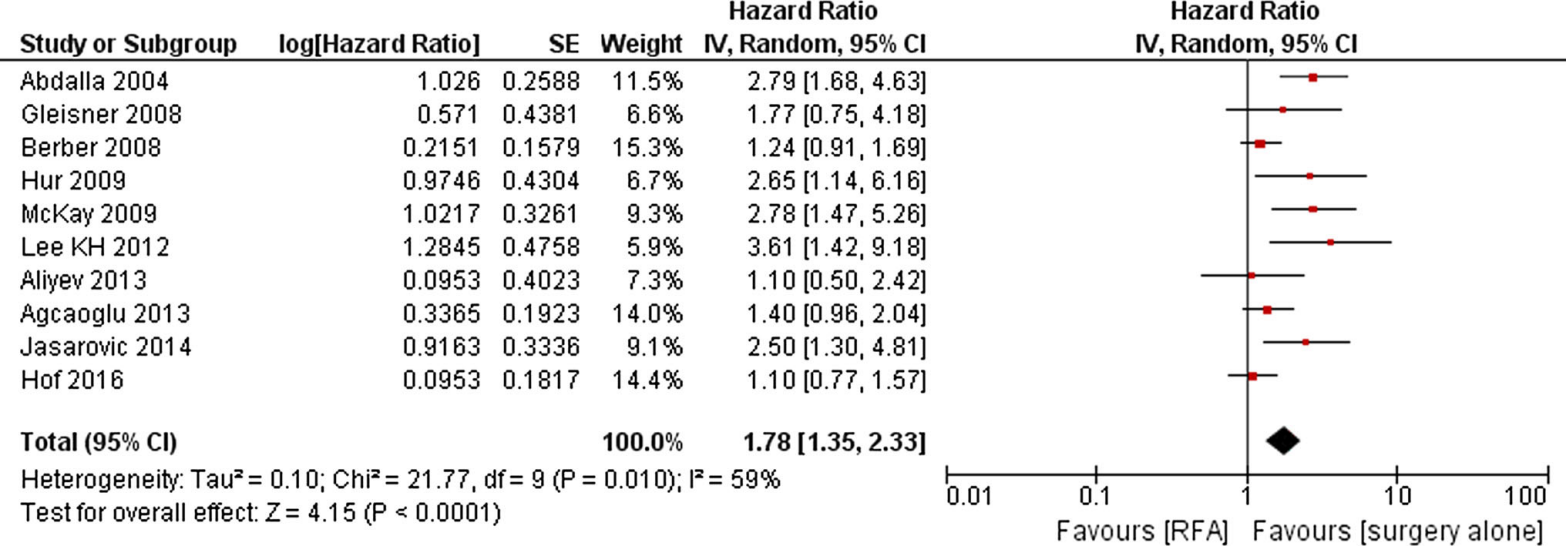
RFA versus PH alone: overall survival (OS)

Ten observational studies (*N* = 1824 reported corrected hazard ratios for OS (Fig. 5) [13, 30, 31, 33–35, 37, 39, 45, 46]. Pooling of the results showed that RFA was associated with an inferior OS (HR = 1.78; 95%CI 1.35–2.33)). Two other studies only reported non-corrected HRs, treatment type was not associated with prognosis based on univariate analysis [41, 47]. Adding these studies to the meta-analysis did not substantially alter the results (HR = 1.62; 95%CI 1.29–2.03).

Five articles allowed for pooling of OS results for solitary metastases. Again, RFA was associated with a less favourable outcome (HR = 1.77; 95%CI 1.18–2.65) [31, 33–35, 39]. The corrected odds ratio as reported by Aloia et al. also showed better results for PH alone (odds ratio 3.22; 95%CI 1.74–5.96) [32].

#### RFA Plus PH Versus PH Alone

**Fig. 6:**
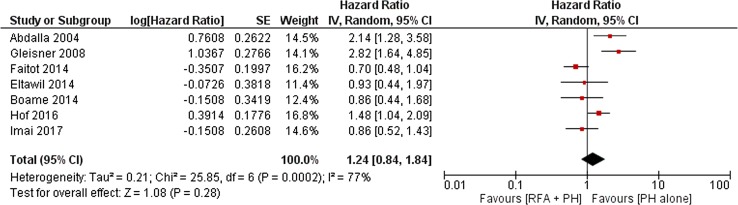
RFA + PH versus PH alone: overall survival (OS)

Seven observational studies (*N* = 1918 reported corrected hazard ratios and allowed for pooling of OS results (Fig. 6) [13, 15, 16, 18, 27, 45, 46]. No significant difference in OS was found (HR = 1.24; 95%CI 0.84–1.84). One other article reported only non-corrected hazard ratios, treatment type was not associated with prognosis based on univariate analysis. Adding this study to the meta-analysis did not meaningfully alter the results: (HR = 1.27; 95%CI 0.90–1.81) [47]. Govindarajan et al. reported the OS for recurrent CRLM, and did not detect a significant difference between PH and PH + RFA for both solitary CRLM (*p* = 0.49) and multiple CRLM (*p* = 0.18) [43].

### Adverse Events and Quality of Life

**Fig. 7:**
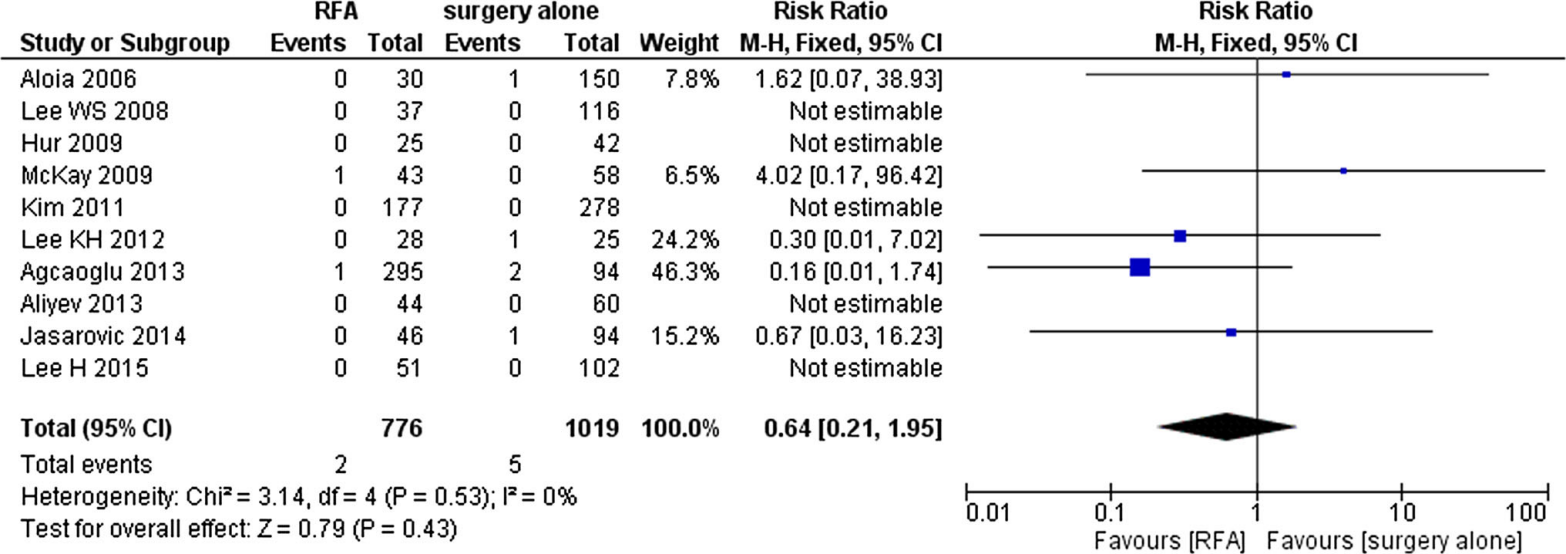
RFA versus PH alone: 30-day mortality

**Fig. 8:**
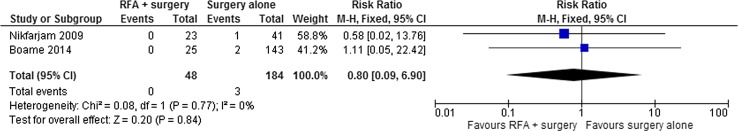
RFA + PH versus PH alone: 60-day mortality

**Fig. 9:**
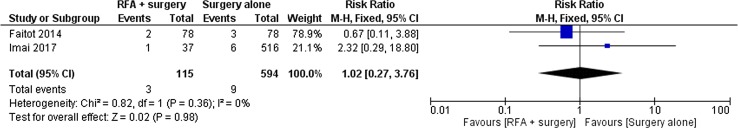
RFA + PH versus PH alone: 90-day mortality

Ruers et al. reported one fatality (sepsis) in the RFA + chemotherapy group [29]. Ten observational studies (*N* = 1795) comparing RFA and PH alone reported post-procedural or 30-day mortality [30–32, 34–39, 47]. Meta-analysis did not show a difference (RR = 0.64; 95%CI 0.21–1.95), although the funnel plot did suggest publication bias (Fig. 7). Of the observational studies comparing RFA + PH and PH alone, one study (*N* = 113) reported 30-day mortality [39], two studies (*N* = 232) reported 60-day mortality [18, 44] (Fig. 8) and two studies (*N* = 709) reported 90-day mortality [15, 27] (Fig. 9). No significant differences were detected (30-day: no events; 60-day: RR = 0.80; 95%CI 0.09–6.90; 90-day: RR = 1.02; 95%Cl 0.27–3.76). Govindarajan et al. reported two deaths within 100-days post-resection in a group of 96 patients versus no deaths in the combination group [43]. Hof et al. only reported the 30-day mortality rate for both interventions (5 of 707 patients) [13].

**Fig. 10:**
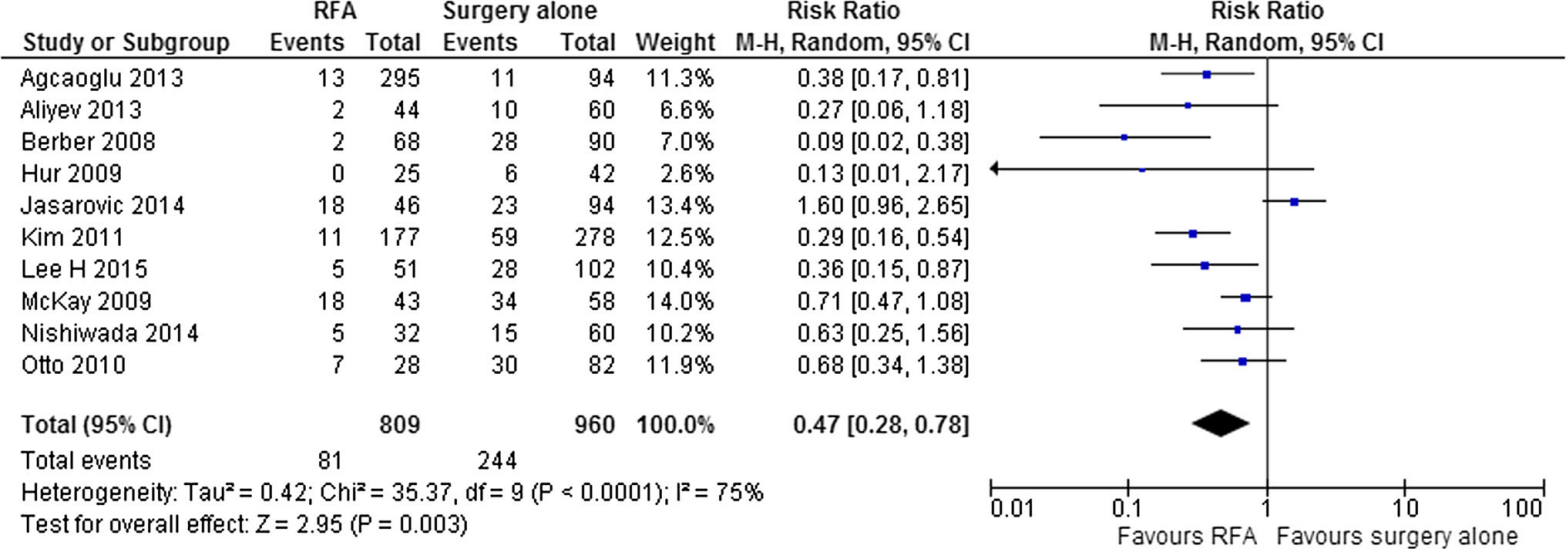
RFA versus PH alone: complication rate

In the EORTC-CLOCC trial, no significant difference in chemotherapy-induced toxicity between the groups was found [29]. In the observational studies comparing RFA and PH alone, complications were more common after PH compared to RFA (10 studies; RR = 0.47; 95%CI 0.28–0.78) (Fig. 10) [30, 31, 33–36, 39–41, 47]. Of the observational studies comparing RFA + PH and PH alone, Faitot et al. reported serious adverse events in 28% after PH (≥ grade 3) versus 13% in the combination group (*p* = 0.017) [15]. Imai et al. reported major complications in 18.6% in the PH alone group (≥ grade 3) versus 22% after PF + RFA (*p* = 0.656) [27]. Kim et al. reported adverse events in 21% after PH (278 patients: 13 haemorrhage, 17 abscesses, 10 wound infections, 8 respiratory failure, 11 ileus) versus 37% in the combination group (27 patients: 3 haemorrhage, 3 abscess, 3 wound infection, 1 respiratory failure) (*p* < 0.001) [47]. Sasaki et al. and Hof et al. didn’t report complications [13, 28].

Ruers et al. reported the effect of RFA on quality of life using EORTC QLQ-C30 questionnaires [29]. With 110 out of 119 patients included in the analysis, overall quality of life decreased 27 points on average after the procedure to partially restore (to 10 points under baseline) prior to starting chemotherapy (4–8 weeks after RFA) and completely restored hereafter. No formal statistical comparison was done.

### Local Progression-Free Survival, Disease-Free Survival and Ablation-Site Recurrence

#### RFA Plus Chemotherapy Versus Chemotherapy Alone

Ruers et al. reported a significantly longer median DFS of 16.8 months (95%CI 11.7–22.1) in the combination group versus 9.9 months (95%CI 9.3–13.7) in the chemotherapy alone group corresponding to a HR of 0.63 (95%CI 0.42–0.95, *p* = 0.025) [29]. The percentage of patients treated for the first progression was comparable between both arms, 37 out of 42 patients (88.1%) in the combination treatment group and 46 out of 53 patients (86.8%) in the systemic treatment group. The long-term results, confirmed an overall DFS favouring RFA + chemotherapy (HR 0.57; 95% CI 0.38–0.85; *p* = 0.005). The 8-year DFS for RFA + chemotherapy versus chemotherapy alone was 22.3% (95%CI 12.7–33.7) versus 2.0% (95%CI 0.2–9.0) [29].

#### RFA Versus PH Alone

**Fig. 11:**
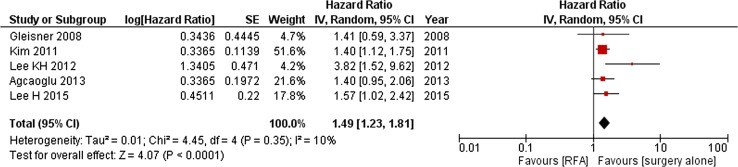
RFA versus PH alone: disease-free survival (DFS)

**Fig. 12:**
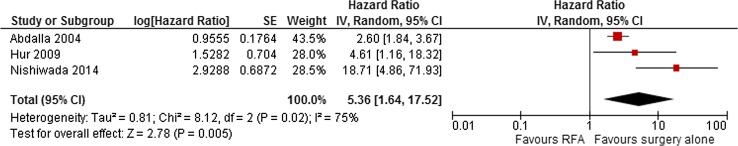
RFA versus PH alone: local progression-free survival (LPFS)

Three and five observational studies (*N* = 406 and *N* = 1253), respectively, reported corrected hazard ratios for DFS [30, 36, 37, 46, 47] and LPFS [34, 40, 45] (Figs. 11, 12). RFA was inferior to PH regarding LPFS and DFS (HR = 5.36 [95%CI 1.64–17.52] and 1.49 [95%CI 1.23–1.81], respectively). One study specifically included patients with solitary CRLM; again PH was superior (HR = 4.61; 95%CI 1.16–18.32) [34]. Most studies did not report corrected data for the number of recurrences. However, Gleisner et al. performed a matched-control and propensity score analysis [46]. At 1 year any disease recurrence was more commonly detected after RFA compared to PH alone (66 vs. 24%; *p* < 0.001) with a high rate of ASR after RFA (41 vs. 2%; *p* < 0.001). Lee et al. also included a propensity score analysis; ASR rate was higher after RFA compared to resection (*p* = 0.021) [36].

#### RFA Plus PH Versus PH Alone

**Fig. 13:**
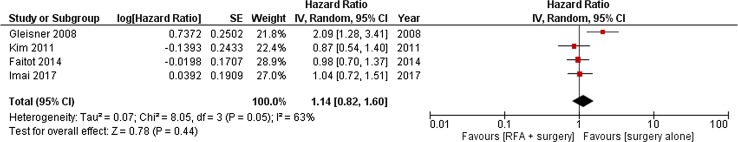
RFA + PH versus PH alone: disease-free survival (DFS)

**Fig. 14:**
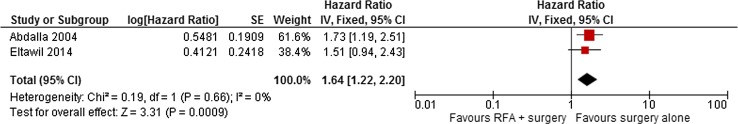
RFA + PH versus PH alone: local progression-free survival (LPFS)

Four and two observational studies (*N* = 1261 and *N* = 465), respectively, reported corrected hazard ratios for DFS [15, 27, 46, 47] and LPFS [16, 45] (Figs. 13, 14). RFA + PH was associated with a poor LPFS compared to PH alone (HR = 1.64; 95%CI 1.22–2.20). No significant difference in DFS between RFA + PH versus PH alone was found (HR = 1.14; 95%CI 0.82–1.60). One study used a matched-control and propensity score analysis which revealed a higher rate of overall and treatment site recurrences after RFA at 1 year (overall 61 vs. 24%; *p* < 0.001 and ASR 10 vs. 2%; *p* < 0.001) [46]. Sasaki et al. and Hof et al. didn’t report corrected hazard ratios for LPFS or DFS [13, 28].

### MWA

**Fig. 15:**
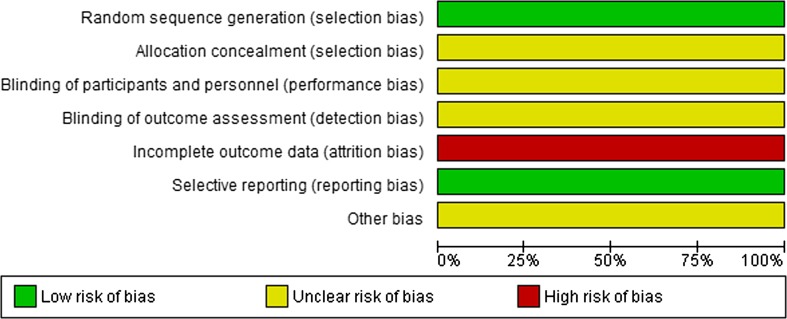
Risk of bias of Shibata et al. [48]

One randomized controlled trial (RCT) compared MWA to hepatectomy in 30 patients with resectable CRLM (Fig. 15) [48]. The absence of an *intention*-*to*-*treat* analysis makes this study at high risk of bias; 25% (10/40) of the randomized patients were not included in the analysis and the precise randomization method remains unclear.

**Fig. 16:**
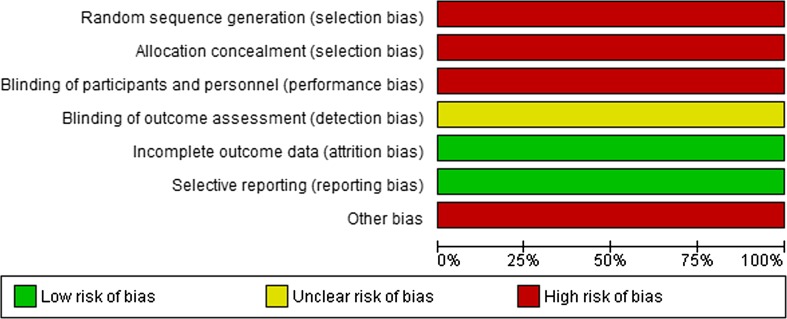
Risk of bias of observational studies for MWA

One observational study compared MWA + PH to PH alone in 53 consecutive patients with at least 5 bilobar CRLM [49]. MWA was performed for unresectable lesions. Another observational study compared a group of 20 patients who underwent MWA for multiple unresectable CRLM with two historical cohorts: 36 patients who had resection and 25 patients who only received systemic treatment [50]. Both studies are at risk of bias due to the absence of a randomization process and the retrospective data collection (Fig. 16).

**Fig. 17:**
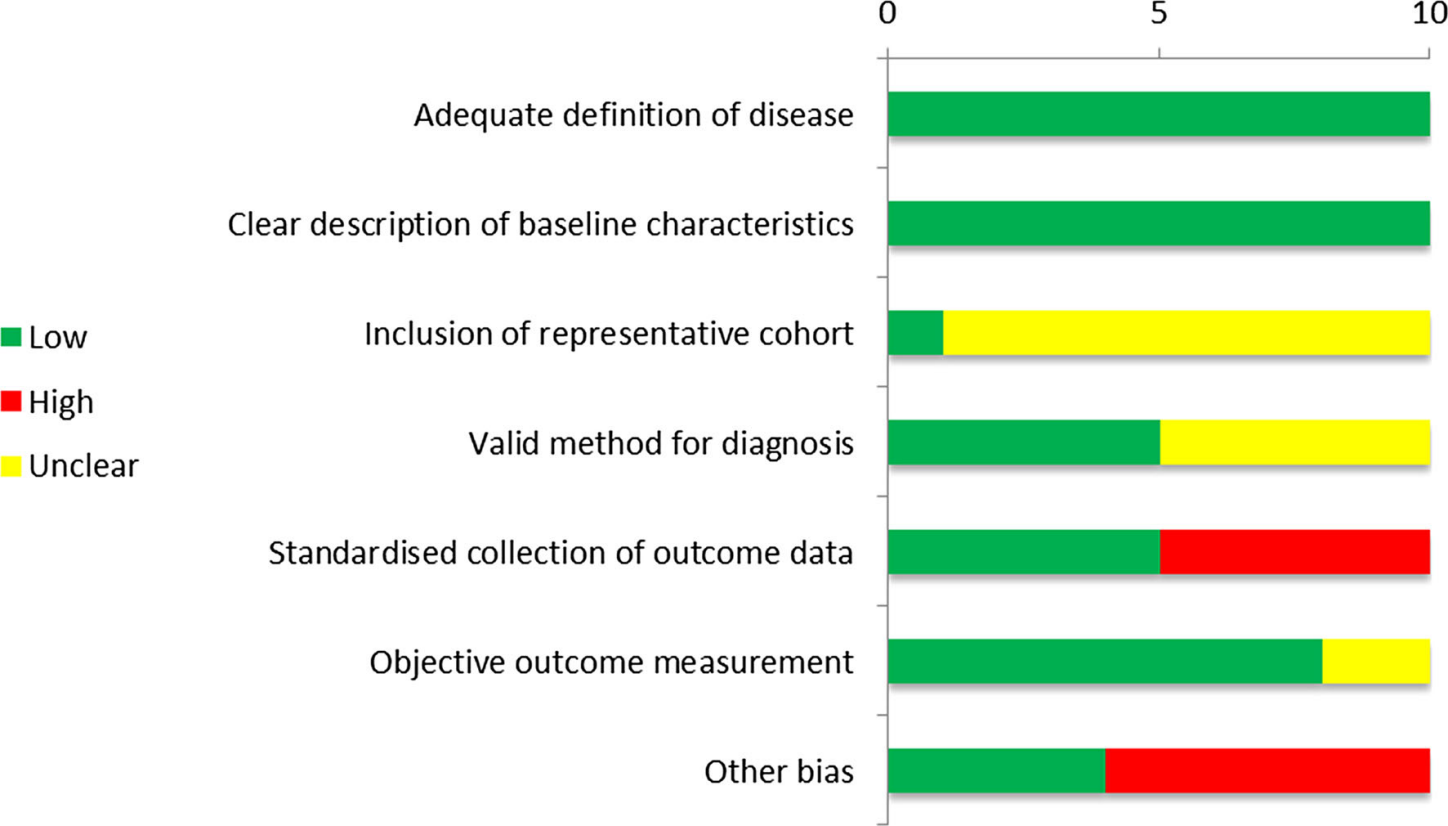
Risk of bias of case series for MWA

Finally, an additional number of ten case series were included (*N* = 689) (Fig. 17) [51–60]. In seven of these, the majority of patients underwent combined resections + MWA [51–55, 57, 59]. Seven studies have a high risk of bias due to retrospective data collection and/or contamination of results after complementary PH [51–55, 57, 59]; in the three other studies risk of bias remains unclear because selection bias cannot be excluded [56, 58, 60]. Only two studies separately reported results for solitary CRLM [56, 58]. Last updated search revealed no extra articles for MWA.

### Overall Survival

Shibata et al. reported a 3-year OS of 23% after hepatectomy and 14% after MWA [48]. Median OS was 25 versus 27 months (*p* = 0.83).

Engstrand et al. reported a 4-year OS of 41% for the MWA group versus 4% in the historical cohort treated with chemotherapy alone [50]. Treatment modality was found to be a prognostic factor in multivariate analysis (HR = 0.56; 95%CI 0.33–0.96). The 4-year OS in the PH alone cohort was 70%, but no formal statistical comparison was reported.

Tanaka et al. did not detect a significant difference in OS between MWA + PH versus PH alone (3-year OS: 50.9 vs. 48.8%) [49]. Median OS was 39 months after PH and 28 months after MWA + PH. In multivariate analysis, MWA was no prognostic factor for OS.

Median OS in five case series ranged between 24 and 36 months [53, 54, 57–59]. The reported 3-,4- and 5-year OS varied between 35–79% [53, 54, 59, 60], 35–58% [52, 55] and 17–18% [53, 59], respectively.

### Mortality, Adverse Events and Quality OF Life

Both Shibata et al. and Tanaka et al. did not detect any mortality after MWA or PH within 60 days after the procedure [48, 49]. Reported mortality in the case series ranged from 0 to 2% [55, 57, 59]. Shibata et al. reported complications in 2/14 patients in the MWA group (1 liver abscess, 1 biliary fistula) and in 3/16 in the PH group (1 intestinal obstruction, 1 biliary fistula, 1 wound infection) (*p* = 0.87) [48]. Tanaka et al. found complications in 6/37 patients undergoing liver PH versus 3/16 in the combination group (no *p* = value reported) [49]. In the case series, the documentation of complications was heterogeneous. Complication rates varied between 0 and 54% [51, 52, 54, 56, 57, 59]. No studies reported the effect on quality of life.

### Disease-Free Survival and Ablation-Site Recurrence

Shibata et al. reported a median DFS of 13.3 months following PH versus 11.3 months following MWA [48]. Tanaka et al. did not detect a significant difference in DFS (4-year DFS: 39 vs. 35%; *p* = 0.86) [49]. After a median follow-up of 21 months, 28/34 (19 in the liver) patients in the PH group had a recurrence versus 11/15 (9 in the liver) in the MWA group after a median follow-up of 19 months.

Eng et al. reported a 3.5-year DFS of 19% [52]. Stattner et al. found a 3-year DFS of 22% for the entire MWA group and 32% for the MWA alone subgroup [59]. Two studies found a median DFS of 8 and 12 months [57, 59]. Groeschl et al. reported a 3- and 5-year DFS of 34 and 9%, respectively [53]. In a second series Groeschl et al. found a 3-year DFS of 0% [54]. Overall recurrence was present in 39–72% [52–54, 57, 59]. In 8 case series ASR varied between 2 and 30% [51–54, 56–59].

### Guidelines

The search for guidelines resulted in 15 references, out of which two were excluded because they were updated by a more recent version [61, 62]. Thirteen references were evaluated based on their full text; all were included and assessed according to the AGREE II instrument (Table 2 online appendix) [63–75]. In 4 guidelines RFA and MWA was not mentioned [63–66]. In 1 guideline RFA was mentioned but without clear recommendations [67]. The American College of Radiology (ACR) guideline does not include specific recommendations, but RFA was described as unsuitable for CRLM, although scientific support for this statement is lacking [68]. The US National Comprehensive Cancer Network (NCCN) guidelines do not provide well-defined recommendations for RFA and MWA, although they do write the following: “The panel does not consider ablation to be a substitute for resection in patients with completely resectable disease. In addition, resection or ablation (either alone or in combination with resection) should be reserved for patients with disease that is completely amenable to local therapy. Use of surgery, ablation, or the combination, with the goal of less-than-complete resection/ablation of all known sites of disease, is not recommended” [69, 70]. References to the EORTC-CLOCC trial and to several observational studies were used to support these statements [3, 29, 46, 76–80]. The European Society for Medical Oncology (ESMO) considers RFA suitable for CRLM < 4 cm if surgery is contra-indicated and refers to the EORTC-CLOCC trial and a systematic review [29, 71, 78]. The UK National Institute for Health and Care Excellence (NICE) guideline considers the current evidence on safety and efficacy adequate to support the use of this procedure in patients unfit or otherwise unsuitable for hepatic resection, or in those who have previously had hepatic resection, provided that normal arrangements are in place for clinical governance, consent and audit [72]. The Scottish Intercollegiate Guidelines network (SIGN) commends that ablation should be considered for CRLM [73, 81]. The Belgian Health Care Knowledge Center (KCE) recommends the use of RFA in combination with PH to preserve sufficient future liver remnant and refers to the NICE, SIGN and CCO guidelines [74]. The most comprehensive recommendations were reported in the Dutch Comprehensive Cancer Centre (IKNL) guideline: thermal ablation cannot be considered a substitute for resection, but represents a suitable treatment option for unresectable CRLM if the goal is a complete eradication of all lesions with curative intent [75]. Percutaneous ablation can be considered for patients who are less suitable for surgery because of high-age, comorbidity, unfavourable location or a history of extensive abdominal surgery. The ablation technique of the first choice is RFA. MWA can be considered a good alternative, especially for lesions in proximity of large blood vessels where heatsink, when heat is carried away by the flowing blood, may enable tumour cells to survive after RFA. IKNL refers to the EORTC-CLOCC trial, the Cochrane review and several observational studies [3, 26, 29, 82–85].

## Discussion

Contradictory to the many available comparative observational studies and case series on thermal ablation for CRLM, the literature to reliably assess its effectiveness compared to chemotherapy and surgery is limited. Although one RCT was identified for RFA [29], GRADE valuation required downgrading the quality of evidence regarding OS. When comparing RFA (± PH) + chemotherapy to chemotherapy alone, quality was downgraded to moderate, especially because both the optimal information size (OIS; number of included patients did not meet sample size) and the reduced relative risk (RRR = 100 * [1 − upper limit of the 95%CI for the HR (0.88)] = 12%) was too low (serious imprecision; Table 1). When comparing RFA + chemotherapy to chemotherapy alone, quality was further downgraded to low, because a substantial part of the ablated patients also underwent PH (serious indirectness). However, the remarkable differences in 8-year OS (8.9 vs. 35.9%) and 8-year DFS (22.3 vs. 2.0%) seem to validate the eradication of all macroscopically visible CRLM and to justify the adoption of thermal ablation for unresectable CRLM for this indication [29]. The very serious risk of bias of the one MWA trial required downgrading to very low-quality evidence.

Comparing PH alone for resectable lesions with RFA for unresectable lesions, RFA was associated with significantly fewer complications but also with an inferior survival. In contrast, RFA in addition to PH for patients with unresectable disease, resulted in a comparable survival to resection alone for patients with resectable disease. In other words, for patients with unresectable disease, in whom palliative chemotherapy used to denote the only treatment option, RFA is able to offer patients a DFS and OS comparable to or approaching that of surgical candidates. Out of the eight studies published after 2012, seven showed a similar OS when comparing ablation (± PH) to PH alone (Figs. 5, 6), which may advert to ablative technique improvements. Although MWA compared to chemotherapy alone was associated with a superior OS for patients with unresectable CRLM, this is based on a single retrospective study at risk of bias due to the unclear randomization process, which seriously demotes quality of evidence [50].

In contrast to RFA, the number of comparative studies for MWA was limited. For this reason, we incorporated more restrictions for the RFA studies, including only RCTs and observational studies that performed either case matching or multivariate analysis for prognostic factors.

The included observational studies were by definition all confounded by indication, since ablation was only performed for unresectable lesions. Reasons for choosing ablation over PH were comorbidity (0–41%), inadequate future liver remnant and/or technical factors such as difficult anatomical location (5–67%), patient’s choice (0–61%) or extrahepatic disease for studies where this was no exclusion criterion (0–19%). Two other methods to adjust for confounding, namely restricting inclusion to patients from one prognostic category (for example bilobar CRLM) or stratification into subgroups were not allowed, because these methods only take one prognostic factor into account. All outcome measures were heterogeneously reported and follow-up periods ranged between 19 and 61 months in observational studies on RFA. The documentation of tumour load and disease status was strongly variable as were the definitions of progression-, recurrence- and disease-free survival.

The reporting of complications was heterogeneous, which is why it is difficult to identify the most frequent complications for thermal ablation. Of the 24 observational studies, only two were published prior to 2008. In recent years, several technical advancements were implemented in the field of RFA, although the same can be assumed for surgical techniques. The impact of these two older reports on the global results is probably limited. For MWA this effect may be greater, because the only RCT was published in 2000 and one of two observational studies in 2006. Although technical factors such as an unfavourable anatomical location were used to choose for thermal ablation, clear definitions for resectability were not provided in any of the included studies, with the exception of Ruers et al., who defined resectability as “the possibility to completely resect all CRLM” [29]. For this reason, subgroup analysis was impossible and the risk for potential confounding by indication remains high. In the thermal ablation studies, the number of procedures necessary to reach local control was heterogeneously reported.

At the time of literature review, there was only one series comparing RFA to MWA for CRLM [86]. Of 243 patients there were no differences regarding OS and ASR between RFA and MWA (*p* = 0.559 and 0.078, respectively), although the complication rate for peribiliary CRLM was higher after MWA (*p* = 0.002).

Conclusions drawn from previous meta-analyses are comparable to ours with regard to patients with resectable CRLM, but differ for patients with unresectable disease. The review from Sutherland et al. [25] (published in 2006) was probably too old to find sufficiently relevant studies. Belinson et al. [2] and Cirocchi et al. [3] concluded: “Evidence from the included studies are insufficient to recommend RFA for a radical oncological treatment of CRLMs”. Gurusamy et al. did not find any RCTs [9]. Bala et al. [1] and Loveman et al. [23] found one RCT for MWA (Shibata et al. [48] published in 2000) and concluded: *“*Evidence is insufficient to show whether microwave coagulation brings any significant benefit in terms of survival or recurrence compared with conventional surgery for CRLM patients*”.* Smith et al. [24] did not assess RFA separately. Pathak et al. [26] were more positive in their conclusions, although their analysis primarily included case series.

The results from this analysis should be judged with caution. Although systematically obtained, there are no guarantees that all available evidence was identified. Furthermore, the inclusion of observational studies increases the risk for publication bias, for which objective indications were detected for the complication rate. Although (for RFA) only studies using randomization, matching or multivariate analysis was included, this does not exclude residual confounding.

To conclude, this article is the first systematic review that supports the widespread adoption of thermal ablation to treat small unresectable CRLM. The (1) recently published long-term survival results from the EORTC-CLOCC trial [29], the (2) comparable survival results after ablation versus resection for the series reported after 2012, the (3) comparable survival after ablation + resection versus resection alone, the (4) potential to induce long-term disease control and the (5) low complication rates all argue in favour of thermal ablation over chemotherapy alone. Further randomized comparisons of thermal ablation with curative intent to current-day palliative chemotherapy alone should therefore be considered unethical. As a consequence, the highest achievable evidence level for unresectable CRLM seems to have been reached.

Although ablation for unresectable CRLM seems inferior to PH for resectable lesions, the lower complication rate combined with the apparent selection bias stresses the need to conduct a randomized controlled trial. Currently, PH for resectable CRLM is being challenged by thermal ablation in a large multicentre, phase III, randomized controlled trial (COLLISION trial; *NCT03088150*). This study assesses overall- and disease-free survival, time to (local) progression, primary and assisted technique efficacy rates, adverse events, quality of life and incremental costs.

## Electronic supplementary material

Below is the link to the electronic supplementary material.

Supplementary material 1 (DOCX 16 kb)

Supplementary material 2 (DOCX 23 kb)

Supplementary material 3 (DOCX 51 kb)

## Abbreviations

ACR: American College of Radiology
ASR: Ablation-site recurrence rate
CI: Confidence interval
CRLM: Colorectal liver metastases
DFS: Disease-free survival
ESMO: European Society for Medical Oncology
GRADE: Grading of Recommendations, Assessment, Development and Evaluation
HR: Hazard ratio
IKNL: Dutch Comprehensive Cancer Centre
KCE: Belgian Health Care Knowledge Center
LPFS: Local progression-free survival
MWA: Microwave ablation
NCCN: National comprehensive cancer network
NICE: National Institute for Health and Care Excellence
OIS: Optimal information size
OS: Overall survival
PH: Partial hepatectomy
QoL: Quality of life
RCT: Randomized controlled trial
RFA: Radiofrequency ablation
RR: Risk ratio
RRR: Reduced relative risk
SIGN: Scottish Intercollegiate Guidelines Network
ZiNL: Dutch National Health Care Institute
