# Supplementary material for: Radiofrequency and Microwave Ablation Compared to Systemic Chemotherapy and to Partial Hepatectomy in the Treatment of Colorectal Liver Metastases: A Systematic Review and Meta-Analysis
Source: Cardiovasc Intervent Radiol. 2018 Apr 17;41(8):1189–204. doi: 10.1007/s00270-018-1959-3 (PMC6021475; doi:10.1007/s00270-018-1959-3)
Supplement: Supplementary file 1 — Supplementary material 1 (DOCX 16 kb) [file 270_2018_1959_MOESM1_ESM.docx]

Table 2 (online appendix): Guidelines from Clearinghouse and International Network Guidelines reviewed according to the AGREE-II instrument.

| **Table 2: Guidelines from Clearinghouse and International Network Guidelines reviewed according to the AGREE-II^*^ instrument.** | | | | | | | | | |
| --- | --- | --- | --- | --- | --- | --- | --- | --- | --- |
|  | **7** | **8** | **9** | **10** | **11** | **12** | **13** | **14** | Domain score |
| ACR 2014 | 2 | 1 | 3 | 6 | 3 | 2 | 1 | 1 | 22.9 |
| ASCRS 2012 (colon) | 6 | 3 | 2 | 2 | 3 | 3 | 2 | 1 | 29.2 |
| ASCRS 2013 (rectum) | 6 | 1.5 | 2 | 2 | 3 | 2.5 | 2 | 1 | 25 |
| CCO 2012 | 6 | 7 | 2,5 | 1.5 | 2.5 | 6 | 7 | 7 | 65.6 |
| ESMO 2012 | 1 | 1 | 1.5 | 3 | 1.5 | 2 | 1 | 2 | 10.4 |
| ESMO 2014 | 1 | 1 | 2 | 1.5 | 2.5 | 2 | 1 | 2 | 10.4 |
| ESMO 2013 | 1 | 1 | 1.5 | 1.5 | 2 | 2.5 | 1 | 2.5 | 10.4 |
| IKNL 2014 | 4 | 3.5 | 3.5 | 5.5 | 5.5 | 6 | 5 | 5 | 62.5 |
| KCE 2014 | 6.5 | 3 | 7 | 6 | 6 | 7 | 6.5 | 7 | 85.4 |
| NCCN 2015 (colon) | 4.5 | 2 | 1.5 | 4.5 | 3.5 | 2 | 1.5 | 7 | 38.5 |
| NCCN 2015 (rectum) | 4.5 | 2 | 1.5 | 4.5 | 4 | 2.5 | 1.5 | 7 | 40.6 |
| NICE 2011 | 7 | 7 | 7 | 6 | 7 | 7 | 5.5 | 6 | 92.7 |
| SIGN 2011 | 4.5 | 3.5 | 3.5 | 4 | 5.5 | 5 | 5 | 4 | 56.3 |
| * A quality score is calculated for each of the six AGREE II domains. The six domain scores are independent and should not be aggregated into a single quality score. Domain scores are calculated by summing up all the scores of the individual items in a domain and by scaling the total as a percentage of the maximum possible score for that domain. Although the domain scores are useful for comparing guidelines and will inform whether a guideline should be recommended for use, the Consortium has not set minimum domain scores or patterns of scores across domains to differentiate between high quality and poor quality guidelines. These decisions should be made by the user and guided by the context in which AGREE II is being used. | | | | | | | | | |
