# Supplementary material for: Radiofrequency and Microwave Ablation Compared to Systemic Chemotherapy and to Partial Hepatectomy in the Treatment of Colorectal Liver Metastases: A Systematic Review and Meta-Analysis
Source: Cardiovasc Intervent Radiol. 2018 Apr 17;41(8):1189–204. doi: 10.1007/s00270-018-1959-3 (PMC6021475; doi:10.1007/s00270-018-1959-3)
Supplement: Supplementary file 2 — Supplementary material 2 (DOCX 23 kb) [file 270_2018_1959_MOESM2_ESM.docx]

Table 3 (online appendix): Search-strategies.

| **Table 3: Online Appendix: Search-strategies.** | |
| --- | --- |
| **MEDLINE and PREMEDLINE (OVID):** | |
| 1 | (colorectal adj5 neoplas$).tw. (3479) |
| 2 | (colorectal adj5 cancer$).tw. (69204) |
| 3 | (colorectal adj5 carcin$).tw. (17703) |
| 4 | (colorectal adj5 tumo$).tw. (10468) |
| 5 | (colorectal adj5 metasta$).tw. (15063) |
| 6 | (colorectal adj5 malig$).tw. (2393) |
| 7 | (colon$ adj5 neoplas$).tw. (3363) |
| 8 | (colon$ adj5 cancer$).tw. (46523) |
| 9 | (colon$ adj5 carcin$).tw. (21414) |
| 10 | (colon$ adj5 tumo$).tw. (16582) |
| 11 | (colon$ adj5 metasta$).tw. (6307) |
| 12 | (colon$ adj5 malig$).tw. (3176) |
| 13 | (rect$ adj5 neoplas$).tw. (738) |
| 14 | (rect$ adj5 cancer$).tw. (20562) |
| 15 | (rect$ adj5 carcin$).tw. (7203) |
| 16 | (rect$ adj5 tumo$).tw. (5197) |
| 17 | (rect$ adj5 metasta$).tw. (1957) |
| 18 | (rect$ adj5 malig$).tw. (1003) |
| 19 | exp Rectal Neoplasms/ (39069) |
| 20 | exp Colorectal Neoplasms/ (156476) |
| 21 | exp Colonic Neoplasms/ (69464) |
| 22 | exp Sigmoid Neoplasms/ (4030) |
| 23 | or/1-22 (208102) |
| 24 | (liver tumo* or liver neoplas* or liver malign* or liver carcinom* or liver metastas*).mp. (136733) |
| 25 | exp Liver Neoplasms/ (131882) |
| 26 | 24 or 25 (140655) |
| 27 | 23 and 26 (16683) |
| 28 | (radiofrequency or radio frequency).mp. (30112) |
| 29 | exp Catheter Ablation/ (23072) |
| 30 | catheter ablation.mp. (25091) |
| 31 | (rfta or RFA).mp. (4135) |
| 32 | (thermoablati* or thermo ablation or thermo destruc* or thermal destruc* or thermo coag* or thermal coag* or electrocoagulation or transvenous ablati*).mp. (12540) |
| 33 | exp Microwaves/ (13443) |
| 34 | microwave ablati*.mp. (675) |
| 35 | MWA.mp. (299) |
| 36 | 28 or 29 or 30 or 31 or 32 or 33 or 34 or 35 (66659) |
| 37 | 27 and 36 (1001) |
| **MEDLINE and PREMEDLINE (OVID): extra (sensitive) search for systematic reviews** | |
| 1 | (colorectal adj5 neoplas$).tw. (3479) |
| 2 | (colorectal adj5 cancer$).tw. (69204) |
| 3 | (colorectal adj5 carcin$).tw. (17703) |
| 4 | (colorectal adj5 tumo$).tw. (10468) |
| 5 | (colorectal adj5 metasta$).tw. (15063) |
| 6 | (colorectal adj5 malig$).tw. (2393) |
| 7 | (colon$ adj5 neoplas$).tw. (3363) |
| 8 | (colon$ adj5 cancer$).tw. (46523) |
| 9 | (colon$ adj5 carcin$).tw. (21414) |
| 10 | (colon$ adj5 tumo$).tw. (16582) |
| 11 | (colon$ adj5 metasta$).tw. (6307) |
| 12 | (colon$ adj5 malig$).tw. (3176) |
| 13 | (rect$ adj5 neoplas$).tw. (738) |
| 14 | (rect$ adj5 cancer$).tw. (20562) |
| 15 | (rect$ adj5 carcin$).tw. (7203) |
| 16 | (rect$ adj5 tumo$).tw. (5197) |
| 17 | (rect$ adj5 metasta$).tw. (1957) |
| 18 | (rect$ adj5 malig$).tw. (1003) |
| 19 | exp Rectal Neoplasms/ (39069) |
| 20 | exp Colorectal Neoplasms/ (156476) |
| 21 | exp Colonic Neoplasms/ (69464) |
| 22 | exp Sigmoid Neoplasms/ (4030) |
| 23 | or/1-22 (208102) |
| 24 | (liver tumo* or liver neoplas* or liver malign* or liver carcinom* or liver metastas*).mp. (136733) |
| 25 | exp Liver Neoplasms/ (131882) |
| 26 | 24 or 25 (140655) |
| 27 | 23 and 26 (16683) |
| 28 | (radiofrequency or radio frequency).mp. (30112) |
| 29 | exp Catheter Ablation/ (23072) |
| 30 | catheter ablation.mp. (25091) |
| 31 | (rfta or RFA).mp. (4135) |
| 32 | (thermoablati* or thermo ablation or thermo destruc* or thermal destruc* or thermo coag* or thermal coag* or electrocoagulation or transvenous ablati*).mp. (12540) |
| 33 | exp Microwaves/ (13443) |
| 34 | microwave ablati*.mp. (675) |
| 35 | MWA.mp. (299) |
| 36 | 28 or 29 or 30 or 31 or 32 or 33 or 34 or 35 (66659) |
| 37 | 27 and 36 (1001) |
| 38 | 26 and 36 (4740) |
| 39 | meta-analysis.mp,pt. or review.pt. or search:.tw. (2213359) |
| 40 | 38 and 39 (995) |
| **MEDLINE and PREMEDLINE (OVID): extra search for liver metastases** | |
| 1 | (colorectal adj5 neoplas$).tw. (3480) |
| 2 | (colorectal adj5 cancer$).tw. (69255) |
| 3 | (colorectal adj5 carcin$).tw. (17707) |
| 4 | (colorectal adj5 tumo$).tw. (10474) |
| 5 | (colorectal adj5 metasta$).tw. (15069) |
| 6 | (colorectal adj5 malig$).tw. (2394) |
| 7 | (colon$ adj5 neoplas$).tw. (3364) |
| 8 | (colon$ adj5 cancer$).tw. (46549) |
| 9 | (colon$ adj5 carcin$).tw. (21419) |
| 10 | (colon$ adj5 tumo$).tw. (16597) |
| 11 | (colon$ adj5 metasta$).tw. (6309) |
| 12 | (colon$ adj5 malig$).tw. (3178) |
| 13 | (rect$ adj5 neoplas$).tw. (739) |
| 14 | (rect$ adj5 cancer$).tw. (20577) |
| 15 | (rect$ adj5 carcin$).tw. (7203) |
| 16 | (rect$ adj5 tumo$).tw. (5198) |
| 17 | (rect$ adj5 metasta$).tw. (1957) |
| 18 | (rect$ adj5 malig$).tw. (1003) |
| 19 | exp Rectal Neoplasms/ (39069) |
| 20 | exp Colorectal Neoplasms/ (156476) |
| 21 | exp Colonic Neoplasms/ (69464) |
| 22 | exp Sigmoid Neoplasms/ (4030) |
| 23 | or/1-22 (208191) |
| 24 | (liver tumo* or liver neoplas* or liver malign* or liver carcinom* or liver metastas*).mp. (136745) |
| 25 | exp Liver Neoplasms/ (131882) |
| 26 | 24 or 25 (140667) |
| 27 | 23 and 26 (16684) |
| 28 | (radiofrequency or radio frequency).mp. (30131) |
| 29 | exp Catheter Ablation/ (23072) |
| 30 | catheter ablation.mp. (25094) |
| 31 | (rfta or RFA).mp. (4138) |
| 32 | (thermoablati* or thermo ablation or thermo destruc* or thermal destruc* or thermo coag* or thermal coag* or electrocoagulation or transvenous ablati*).mp. (12541) |
| 33 | exp Microwaves/ (13443) |
| 34 | microwave ablati*.mp. (676) |
| 35 | MWA.mp. (300) |
| 36 | 28 or 29 or 30 or 31 or 32 or 33 or 34 or 35 (66681) |
| 37 | 27 and 36 (1001) |
| 38 | Ablation Techniques/ (1106) |
| 39 | Liver Neoplasms/sc [Secondary] (25016) |
| 40 | liver metasta*.mp. (18097) |
| 41 | 39 or 40 (32100) |
| 42 | 36 or 38 (67493) |
| 43 | 41 and 42 (1592) |
| 44 | 43 not 37 (677) |
| 45 | (colorectal adj5 neoplas$).tw. (3480) |
| 46 | (colorectal adj5 cancer$).tw. (69255) |
| 47 | (colorectal adj5 carcin$).tw. (17707) |
| 48 | (colorectal adj5 tumo$).tw. (10474) |
| 49 | (colorectal adj5 metasta$).tw. (15069) |
| 50 | (colorectal adj5 malig$).tw. (2394) |
| 51 | (colon$ adj5 neoplas$).tw. (3364) |
| 52 | (colon$ adj5 cancer$).tw. (46549) |
| 53 | (colon$ adj5 carcin$).tw. (21419) |
| 54 | (colon$ adj5 tumo$).tw. (16597) |
| 55 | (colon$ adj5 metasta$).tw. (6309) |
| 56 | (colon$ adj5 malig$).tw. (3178) |
| 57 | (rect$ adj5 neoplas$).tw. (739) |
| 58 | (rect$ adj5 cancer$).tw. (20577) |
| 59 | (rect$ adj5 carcin$).tw. (7203) |
| **EMBASE** | |
| 1 | ((colorectal OR colon* OR rect*) NEAR/5 (neoplas* OR cancer* OR carcin* OR tumo* OR metasta* OR malig*)):ab,ti |
| 2 | colon cancer'/exp OR 'rectum cancer'/exp |
| 3 | #1 OR #3 |
| 4 | (liver NEAR/5 (tumo* OR neoplas* OR malign* OR carcinom* OR metastas*)):ab,ti |
| 5 | liver metastasis'/exp |
| 6 | #4 OR #5 |
| 7 | #3 AND #6 |
| 8 | radiofrequency ablation'/exp OR 'radiofrequency ablation device'/exp OR 'catheter ablation'/exp OR 'microwave therapy'/exp |
| 9 | radiofrequency:ab,ti OR 'radio frequency':ab,ti |
| 10 | catheter ablation':ab,ti |
| 11 | rfta:ab,ti OR rfa:ab,ti |
| 12 | thermoablati*:ab,ti OR 'thermo ablation':ab,ti OR (thermo NEAR/1 destruc*):ab,ti OR (thermal NEAR/1 destruc*):ab,ti OR (thermo NEAR/1 coag*):ab,ti OR (thermal NEAR/1 coag*):ab,ti OR electrocoagulation:ab,ti OR (transvenous NEAR/1 ablati*):ab,ti |
| 13 | (microwave NEAR/1 ablati*):ab,ti |
| 14 | mwa:ab,ti |
| 15 | #8 OR #9 OR #10 OR #11 OR #12 OR #13 OR #14 |
| 16 | #7 AND #15 |
| 17 | #16 AND ([article]/lim OR [article in press]/lim OR [review]/lim) AND ([dutch]/lim OR [english]/lim OR [french]/lim) |
| **COCHRANE LIBRARY** | |
| 1 | ((colorectal or rect* or colon*) and (neoplas* or cancer* or carcin* or tumo* or metasta* or malig*)):ti,ab |
| 2 | MeSH descriptor: [Rectal Neoplasms] 1 tree(s) exploded |
| 3 | MeSH descriptor: [Colorectal Neoplasms] 1 tree(s) exploded |
| 4 | MeSH descriptor: [Colonic Neoplasms] 1 tree(s) exploded |
| 5 | MeSH descriptor: [Sigmoid Neoplasms] 1 tree(s) exploded |
| 6 | #1 or #2 or #3 or #4 or #5 |
| 7 | (liver and (tumo* or neoplas* or malign* or carcinom* or metastas*)):ti,ab |
| 8 | MeSH descriptor: [Liver Neoplasms] 1 tree(s) exploded |
| 9 | #7 or #8 |
| 10 | #6 and #9 |
| 11 | (radiofrequency or radio frequency):ti,ab |
| 12 | (catheter ablation):ti,ab |
| 13 | MeSH descriptor: [Catheter Ablation] 1 tree(s) exploded |
| 14 | (rfta or RFA):ti,ab |
| 15 | (thermoablati* or thermo ablation or thermo destruc* or thermal destruc* or thermo coag* or thermal coag* or electrocoagulation or transvenous ablati*):ti,ab |
| 16 | (microwave ablati*):ti,ab |
| 17 | MWA:ti,ab |
| 18 | MeSH descriptor: [Microwaves] 1 tree(s) exploded |
| 19 | #11 or #12 or #13 or #14 or #15 or #16 or #17 or #18 |
| 20 | #10 and #19 |
