# Supplementary material for: Radiofrequency and Microwave Ablation Compared to Systemic Chemotherapy and to Partial Hepatectomy in the Treatment of Colorectal Liver Metastases: A Systematic Review and Meta-Analysis
Source: Cardiovasc Intervent Radiol. 2018 Apr 17;41(8):1189–204. doi: 10.1007/s00270-018-1959-3 (PMC6021475; doi:10.1007/s00270-018-1959-3)
Supplement: Supplementary file 3 — Supplementary material 3 (DOCX 51 kb) [file 270_2018_1959_MOESM3_ESM.docx]

Table 4 (online appendix): List of excluded studies.

| Table 4: List of excluded studies. | |
| --- | --- |
| Study | Reason |
| Abbott DE, Sohn VY, Hanseman D, Curley SA. Cost-effectiveness of simultaneous resection and RFA versus 2-stage hepatectomy for bilobar colorectal liver metastases. J Surg Oncol. 2014;109(6):516-20. | Cost-effectiveness study |
| Abitabile P, Hartl U, Lange J, Maurer CA. Radiofrequency ablation permits an effective treatment for colorectal liver metastasis. Eur J Surg Oncol. 2007;33(1):67-71. | Single cohort, no comparison |
| Ahmad A, Chen SL, Kavanagh MA, Allegra DP, Bilchik AJ. Radiofrequency ablation of hepatic metastases from colorectal cancer: are newer generation probes better? Am Surg. 2006;72(10):875-9. | Wrong comparator: RFA vs. RFA |
| Aksoy E, Aliyev S, Taskin HE, Birsen O, Mitchell J, Siperstein A, et al. Clinical scenarios associated with local recurrence after laparoscopic radiofrequency thermal ablation of colorectal liver metastases. Surgery. 2013;154(4):748-52; discussion 52-4. | Single cohort, no comparison |
| Al-Asfoor A, Fedorowicz Z, Lodge M. Resection versus no intervention or other surgical interventions for colorectal cancer liver metastases. Cochrane Database of Systematic Reviews. 2008;2(2):CD006039. | Wrong comparison: only one study included in this review which compares cryosurgery with surgery |
| Amerongen MJ, Jenniskens SFM, van den Boezem P, et al. Radiofrequency ablation compared to surgical resection for curative treatment of patients with colorectal liver metastasis: a meta-analysis. | Poster presentation |
| Amersi FF, McElrath-Garza A, Ahmad A, Zogakis T, Allegra DP, Krasne R, et al. Long-term survival after radiofrequency ablation of complex unresectable liver tumors. Arch Surg. 2006;141(6):581-7; discussion 7-8. | Single cohort, no comparison |
| Andreano A, Galimberti S, Franza E, Knavel EM, Sironi S, Lee FT, et al. Percutaneous microwave ablation of hepatic tumors: prospective evaluation of postablation syndrome and postprocedural pain. J Vasc Interv Radiol. 2014;25(1):97-105.e1-2. | No separate results for CRLM |
| Ayav A, Germain A, Marchal F, Tierris I, Laurent V, Bazin C, et al. Radiofrequency ablation of unresectable liver tumors: factors associated with incomplete ablation or local recurrence. Am J Surg. 2010;200(4):435-9. | Wrong comparator: RFA vs. RFA |
| Azzarello G, Lanteri R, Gresta S, Rapisarda C, Racalbuto A, Di Cataldo A, et al. Thermic ablation with RF of liver metastases from colorectal cancer. Hepato Gastroenterology. 2003;50(2). | Single cohort, no comparison |
| Babawale SN, Jensen TM, Frokjaer JB. Long-term survival following radiofrequency ablation of colorectal liver metastases: A retrospective study. World J Gastrointest Surg. 2015;7(3):33-8. | Single cohort, no comparison |
| Bachar GN, Greif F, Mor E, Tur-Kaspa R, Belenky A. Radiofrequency ablation for the management of liver tumors. Isr Med Assoc J. 2003;5(7):496-500. | Single cohort, no comparison |
| Bai H, Huang X, Jing L, Zeng Q, Han L. The effect of radiofrequency ablation vs. Liver resection on survival outcome of colorectal liver metastases (CRLM): A meta-analysis. Hepato Gastroenterology. 2015;62(138):373-7. | No quality appraisal of included studies |
| Bai H, Huangz X, Jing L, Zeng Q, Han L. The effect of radiofrequency ablation vs. liver resection on survival outcome of colorectal liver metastases (CRLM): a meta-analysis. Hepatogastroenterology. 2015;62(138):373-7. | Double |
| Baldwin K, Katz SC, Rubin A, Somasundar P. Bipolar radiofrequency ablation of liver tumors: technical experience and interval follow-up in 22 patients with 33 ablations. J Surg Oncol. 2012;106(7):905-10. | Single cohort, no comparison |
| Bale R, Widmann G, Schullian P, Haidu M, Pall G, Klaus A, et al. Percutaneous stereotactic radiofrequency ablation of colorectal liver metastases. Eur Radiol. 2012;22(4):930-7. | Single cohort, no comparison |
| Basdanis G, Michalopoulos A, Papadopoulos V, Tzeveleki I, Efthimiadis C, Kosmidis C, et al. Clinical short-term results of radiofrequency ablation in patients with liver metastases from colorectal cancer. Techniques in Coloproctology. 2004;8(1). | Single cohort, no comparison |
| Berber E, Herceg NL, Casto KJ, Siperstein AE. Laparoscopic radiofrequency ablation of hepatic tumors: prospective clinical evaluation of ablation size comparing two treatment algorithms. Surg Endosc. 2004;18(3):390-6. | Single cohort, no comparison |
| Berber E, Pelley R, Siperstein AE. Predictors of survival after radiofrequency thermal ablation of colorectal cancer metastases to the liver: a prospective study. J Clin Oncol. 2005;23(7):1358-64. | Single cohort, no comparison |
| Berber E, Senagore A, Remzi F, Rogers S, Herceg N, Casto K, et al. Laparoscopic radiofrequency ablation of liver tumors combined with colorectal procedures. Surg Laparosc Endosc Percutan Tech. 2004;14(4):186-90. | No matching |
| Berber E, Siperstein A. Local recurrence after laparoscopic radiofrequency ablation of liver tumors: an analysis of 1032 tumors. Ann Surg Oncol. 2008;15(10):2757-64. | Single cohort, no comparison |
| Berber E, Siperstein AE. Perioperative outcome after laparoscopic radiofrequency ablation of liver tumors: an analysis of 521 cases. Surg Endosc. 2007;21(4):613-8. | Single cohort, no comparison |
| Bertot LC, Sato M, Tateishi R, Yoshida H, Koike K. Mortality and complication rates of percutaneous ablative techniques for the treatment of liver tumors: a systematic review. Eur Radiol. 2011;21(12):2584-96. | No separate results for CRLM |
| Bhardwaj N, Strickland AD, Ahmad F, Dennison AR, Lloyd DM. Liver ablation techniques: a review. Surg Endosc. 2010;24(2):254-65. | No quality appraisal of included studies |
| Bilchik AJ, Rose DM, Allegra DP, Bostick PJ, Hsueh E, Morton DL. Radiofrequency ablation: a minimally invasive technique with multiple applications. Cancer J Sci Am. 1999;5(6):356-61. | No separate results for CRLM |
| Birsen O, Aliyev S, Aksoy E, Taskin H.E, Akyuz M, Karabulut K, et al. A critical analysis of postoperative morbidity and mortality after laparoscopic radiofrequency ablation of liver tumors. Ann. Surg. Oncol. 2014;21(6):1834-40. | Single cohort, no comparison |
| Bleicher RJ, Allegra DP, Nora DT, Wood TF, Foshag LJ, Bilchik AJ. Radiofrequency ablation in 447 complex unresectable liver tumors: lessons learned. Ann Surg Oncol. 2003;10(1):52-8. | Single cohort, no comparison |
| Blokhuis TJ, van der Schaaf MC, van den Tol MP, Comans EFI, Manoliu RA, van der Sijp JRM. Results of radio frequency ablation of primary and secondary liver tumors: long-term follow-up with computed tomography and positron emission tomography-18F-deoxyfluoroglucose scanning. Scandinavian Journal of Gastroenterology Supplement. 2004;241(241):93-7. | Single cohort, no comparison |
| Bloomston M, Binitie O, Fraiji E, Murr M, Zervos E, Goldin S, et al. Transcatheter arterial chemoembolization with or without radiofrequency ablation in the management of patients with advanced hepatic malignancy. Am Surg. 2002;68(9):827-31. | No separate results for CRLM |
| Blusse van Oud-Alblas M, Fioole B, Jansen MC, van Duijnhoven FH, van Hillegersberg R, Rijken AM, et al. Radiofrequency ablation of colorectal metastases to the liver: results since the first application in the Netherlands. Ned Tijdschr Geneeskd. 2008;152(15):880-6. | Wrong comparison |
| Bonastre J, De Baere T, Elias D, Evrard S, Rouanet P, Bazin C, et al. Cost of radiofrequency ablation in the treatment of hepatic malignancies. Gastroenterol Clin Biol. 2007;31(10):828-35. | Wrong comparison; cost study |
| Boutros C, Somasundar P, Garrean S, Saied A, Espat NJ. Microwave coagulation therapy for hepatic tumors: review of the literature and critical analysis. Surg Oncol. 2010;19(1):e22-32. | Narrative review |
| Brandi G, de Lorenzo S, Nannini M, curti S, et al. Adjuvant chemotherapy for resected colorectal cancer metastases: literature review and meta-analysis. World J gastroenterol. 2016;22(2):519-533 | Wrong comparison |
| Buell JF, Thomas MT, Rudich S, Marvin M, Nagubandi R, Ravindra KV, et al. Experience with more than 500 minimally invasive hepatic procedures. Ann Surg. 2008;248(3):475-86. | Wrong comparison |
| Buscarini E, Buscarini L. Radiofrequency thermal ablation with expandable needle of focal liver malignancies: complication report. Eur Radiol. 2004;14(1):31-7. | Single cohort, no comparison |
| Carrafiello G, Lagana D, Ianniello A, Dionigi G, Novario R, Recaldini C, et al. Post-radiofrequency ablation syndrome after percutaneous radiofrequency of abdominal tumours: one centre experience and review of published works. Australas Radiol. 2007;51(6):550-4. | Single cohort, no comparison |
| Casaril A, Abu Hilal M, Harb A, Campagnaro T, Mansueto G, Nicoli N. The safety of radiofrequency thermal ablation in the treatment of liver malignancies. Eur J Surg Oncol. 2008;34(6):668-72. | Single cohort, no comparison |
| Cassera MA, Potter KW, Ujiki MB, Swanstrom LL, Hansen PD. Computed tomography (CT)-guided versus laparoscopic radiofrequency ablation: a single-institution comparison of morbidity rates and hospital costs. Surg Endosc. 2011;25(4):1088-95. | Single cohort, no comparison |
| Cazzato RL, Buy X, Alberti N, Fonck M, Grasso RF, Palussiere J. Flat-panel cone-beam CT-guided radiofrequency ablation of very small (< 1.5 cm) liver tumors: technical note on a preliminary experience. Cardiovasc Intervent Radiol. 2015;38(1):206-12. | Single cohort, no comparison |
| Chan RP, Asch M, Kachura J, Ho C-S, Greig P, Langer B, et al. Radiofrequency ablation of malignant hepatic neoplasms. Can Assoc Radiol J. 2002;53(5):272-8. | Single cohort, no comparison |
| Charalampopoulos A, Macheras A, Misiakos E, Batistatou A, Peschos D, Fotiadis K, et al. Thoracoabdominal wall tumour seeding after percutaneous radiofrequency ablation for recurrent colorectal liver metastatic lesion: a case report with a brief literature review. Acta Gastroenterol Belg. 2007;70(2):239-42. | Case report |
| Chen M-H, Yang W, Yan K, Gao W, Dai Y, Wang Y-B, et al. Treatment efficacy of radiofrequency ablation of 338 patients with hepatic malignant tumor and the relevant complications. World J Gastroenterol. 2005;11(40):6395-401. | Single cohort, no comparison |
| Chen M-H, Yang W, Yan K, Zou M-W, Solbiati L, Liu J-B, et al. Large liver tumors: protocol for radiofrequency ablation and its clinical application in 110 patients--mathematic model, overlapping mode, and electrode placement process. Radiology. 2004;232(1):260-71. | Single cohort, no comparison |
| Chen T-M, Huang P-T, Lin L-F, Tung J-N. Major complications of ultrasound-guided percutaneous radiofrequency ablations for liver malignancies: single center experience. J Gastroenterol Hepatol. 2008;23(8 Pt 2):e445-50. | Single cohort, no comparison |
| Cheng J, Glasgow RE, O'Rourke RW, Swanstrom LL, Hansen PD. Laparoscopic radiofrequency ablation and hepatic artery infusion pump placement in the evolving treatment of colorectal hepatic metastases.[Erratum appears in Surg Endosc. 2003 Apr;17(4):669]. Surg Endosc. 2003;17(1):61-7. | No multivariate analysis or matching |
| Chhabra DG, Shah RC, Parikh V, Jagannath P. Radiofrequency ablation of liver tumors: experience with open and percutaneous approach. Indian J Gastroenterol. 2006;25(2):66-70. | Wrong comparison |
| Chiou Y.-Y, Chou Y.-H, Chiang J.-H, Wang H.-K, Chang C.-Y. Percutaneous ultrasound-guided radiofrequency ablation of colorectal liver metastases. Chin. J. Radiol. 2005;30(3):153-8. | Single cohort, no comparison |
| Chopra S, Dodd GD, 3rd, Chanin MP, Chintapalli KN. Radiofrequency ablation of hepatic tumors adjacent to the gallbladder: feasibility and safety. AJR Am J Roentgenol. 2003;American Journal of Roentgenology. 180(3):697-701. | Single cohort, no comparison |
| Chopra S, Dodd GD, 3rd, Chintapalli KN, Leyendecker JR, Karahan OI, Rhim H. Tumor recurrence after radiofrequency thermal ablation of hepatic tumors: spectrum of findings on dual-phase contrast-enhanced CT. AJR Am J Roentgenol. 2001;American Journal of Roentgenology. 177(2):381-7. | Single cohort, no comparison |
| Chow DHF, Sinn LHY, Ng KK, Lam CM, Yuen J, Fan ST, et al. Radiofrequency ablation for hepatocellular carcinoma and metastatic liver tumors: a comparative study. J Surg Oncol. 2006;94(7):565-71. | Comparison group is patients with HCC |
| Choy PYG, Koea J, McCall J, Holden A, Osbourne M. The role of radiofrequency ablation in the treatment of primary and metastatic tumours of the liver: initial lessons learned. N Z Med J. 2002;115(1159):U128. | Wrong comparison |
| Crucitti A, Danza FM, Antinori A, Vincenzo A, Pirulli PGV, Bock E, et al. Radiofrequency thermal ablation (RFA) of liver tumors: percutaneous and open surgical approaches. J Exp Clin Cancer Res. 2003;22(4 Suppl):191-5. | Wrong comparison |
| Crucitti A, Danza FM, Pirulli PGV, Antinori A, Antonacci V, La Greca A, et al. Radiofrequency thermal ablation (RFA) of liver tumors: open surgical or percutaneous approach? Journal of Chemotherapy. 2004;5:82-5. | Wrong comparison |
| Curley SA, Izzo F, Delrio P, Ellis LM, Granchi J, Vallone P, et al. Radiofrequency ablation of unresectable primary and metastatic hepatic malignancies: results in 123 patients. Ann Surg. 1999;230(1):1-8. | Wrong comparison |
| Curley SA, Marra P, Beaty K, Ellis LM, Vauthey JN, Abdalla EK, et al. Early and late complications after radiofrequency ablation of malignant liver tumors in 608 patients. Ann Surg. 2004;239(4):450-8. | Wrong comparison |
| Curley SA. Radiofrequency ablation of malignant liver tumors. Ann Surg Oncol. 2003;10(4):338-47. | Wrong comparison |
| Curley SA. Radiofrequency ablation versus resection for resectable colorectal liver metastases: time for a randomized trial? Ann Surg Oncol. 2008;15(1):11-3. | Editorial |
| Cuschieri A, Bracken J, Boni L. Initial experience with laparoscopic ultrasound-guided radiofrequency thermal ablation of hepatic tumours. Endoscopy. 1999;31(4):318-21. | Single cohort, no comparison |
| de Baere T, Elias D, Dromain C, Din MG, Kuoch V, Ducreux M, et al. Radiofrequency ablation of 100 hepatic metastases with a mean follow-up of more than 1 year. AJR Am J Roentgenol. 2000;American Journal of Roentgenology. 175(6):1619-25. | Single cohort, no comparison |
| De Baere T, Elias D, Ducreux M, Dromain C, Kuach V, Gamal El Din M, et al. Percutaneous radiofrequency ablation of hepatic metastases. Preliminary experience. Gastroenterol Clin Biol. 1999;23(11):1128-33. | French |
| de Baere T, Risse O, Kuoch V, Dromain C, Sengel C, Smayra T, et al. Adverse events during radiofrequency treatment of 582 hepatic tumors. AJR Am J Roentgenol. 2003;American Journal of Roentgenology. 181(3):695-700. | Wrong comparison |
| de Jong MC, van Vledder MG, Ribero D, Hubert C, Gigot J-F, Choti MA, et al. Therapeutic efficacy of combined intraoperative ablation and resection for colorectal liver metastases: an international, multi-institutional analysis. J Gastrointest Surg. 2011;15(2):336-44. | Single cohort, no comparison |
| de Leur K, Huisman F, Gobardhan PD, Kint PAM, Wijsman JH, Ten Tije AJ, et al. Treatment of colorectal liver metastasis at a regional hospital versus a university medical centre. Nederlands Tijdschrift voor Geneeskunde. 2013;157(34):A6417. | No multivariate analysis or matching |
| de Meijer VE, Verhoef C, Kuiper JW, Alwayn IPJ, Kazemier G, Ijzermans JNM. Radiofrequency ablation in patients with primary and secondary hepatic malignancies. J Gastrointest Surg. 2006;10(7):960-73. | Single cohort, no comparison |
| Decadt B, Siriwardena A.K. Radiofrequency ablation of liver tumours: Systematic review. Lancet Oncol. 2004;5(9):550-60. | No quality appraisal of included studies |
| Desolneux G, Vara J, Razafindratsira T, Isambert M, Brouste V, McKelvie-Sebileau P, et al. Patterns of complications following intraoperative radiofrequency ablation for liver metastases. HPB. 2014;16(11):1002-8. | Wrong comparison |
| Dexiang Z, Li R, Ye W, Haifu W, Yunshi Z, Qinghai Y, et al. Outcome of patients with colorectal liver metastasis: analysis of 1,613 consecutive cases. Ann Surg Oncol. 2012;19(9):2860-8. | Wrong comparison |
| Eisele RM, Neumann U, Neuhaus P, Schumacher G. Open surgical is superior to percutaneous access for radiofrequency ablation of hepatic metastases. World J Surg. 2009;33(4):804-11. | Wrong comparison |
| Eisele RM, Zhukowa J, Chopra S, Schmidt SC, Neumann U, Pratschke J, et al. Results of liver resection in combination with radiofrequency ablation for hepatic malignancies. Eur J Surg Oncol. 2010;36(3):269-74. | Single cohort, no comparison |
| Elias D, Baton O, Sideris L, Boige V, Malka D, Liberale G, et al. Hepatectomy plus intraoperative radiofrequency ablation and chemotherapy to treat technically unresectable multiple colorectal liver metastases. J Surg Oncol. 2005;90(1):36-42. | Single cohort, no comparison |
| Elias D, Baton O, Sideris L, Matsuhisa T, Pocard M, Lasser P. Local recurrences after intraoperative radiofrequency ablation of liver metastases: a comparative study with anatomic and wedge resections. Ann Surg Oncol. 2004;11(5):500-5. | No separate results for CRLM |
| Elias D, Baton O, Sideris L, Matsuhisa T, Pocard M, Lasser P. Local recurrences after intraoperative radiofrequency ablation of liver metastases: A comparative study with anatomic and wedge resections. Ann. Surg. Oncol. 2004;11(5):500-5. | Double |
| Elias D, De Baere T, Smayra T, Ouellet JF, Roche A, Lasser P. Percutaneous radiofrequency thermoablation as an alternative to surgery for treatment of liver tumour recurrence after hepatectomy. Br J Surg. 2002;89(6):752-6. | Single cohort, no comparison |
| Elias D, Goharin A, El Otmany A, Taieb J, Duvillard P, Lasser P, et al. Usefulness of intraoperative radiofrequency thermoablation of liver tumours associated or not with hepatectomy. Eur J Surg Oncol. 2000;26(8):763-9. | No separate results for CRLM |
| Engstrand J, Nilsson H, Jansson A, Jonas E, Freedman J. Fate of necrotic volume after microwave ablation of multiple liver metastases. Hepatogastroenterology. 2015;62(137):108-10. | Wrong outcomes |
| Erce C, Parks RW. Interstitial ablative techniques for hepatic tumours. Br J Surg. 2003;90(3):272-89. | Narrative review |
| Evrard S, Becouarn Y, Fonck M, Brunet R, Mathoulin-Pelissier S, Picot V. Surgical treatment of liver metastases by radiofrequency ablation, resection, or in combination. Eur J Surg Oncol. 2004;30(4):399-406. | Single cohort, no comparison |
| Evrard S, Rivoire M, Arnaud JP, Lermite E, Bellera C, Fonck M, et al. Unresectable colorectal cancer liver metastases treated by intraoperative radiofrequency ablation with or without resection. Br J Surg. 2012;99(4):558-65. | Wrong comparison |
| Fedorowicz Z, Lodge M, Al-asfoor A, Carter B. Resection versus no intervention or other surgical interventions for colorectal cancer liver metastases. Cochrane Database of Systematic Reviews. 2008(2). | Outdated, update from 2011 exist |
| Fisichella R, Sparta D, Berretta S. Combined microwave thermal ablation and liver resection for single step treatment of otherwise unresectable colorectal liver metastases; a monoistitutional experiences. European Review for Medical & Pharmacological Sciences. 2015;19(2):180-1. | Letter |
| Fox JP, Gustafson J, Desai MM, Hellan M, Thambi-Pillai T, Ouellette J. Short-term outcomes of ablation therapy for hepatic tumors: evidence from the 2006-2009 nationwide inpatient sample. Ann Surg Oncol. 2012;19(12):3677-86. | Wrong comparison |
| Frezza EE, Wachtel MS, Barragan B, Chiriva-Internati M, Cobos E. The role of radiofrequency ablation in multiple liver metastases to debulk the tumor: a pilot study before alternative therapies. J Laparoendosc Adv Surg Tech A. 2007;Part A. 17(3):282-4. | Single cohort, no comparison |
| Garrean S, Hering J, Helton WS, Espat NJ. A primer on transarterial, chemical, and thermal ablative therapies for hepatic tumors. Am J Surg. 2007;194(1):79-88. | No quality appraisal of included studies |
| Garrean S, Hering J, Saied A, Helton WS, Espat NJ. Radiofrequency ablation of primary and metastatic liver tumors: a critical review of the literature. Am J Surg. 2008;195(4):508-20. | Narrative review |
| Gazelle GS, McMahon PM, Beinfeld MT, Halpern EF, Weinstein MC. Metastatic colorectal carcinoma: cost-effectiveness of percutaneous radiofrequency ablation versus that of hepatic resection. Radiology. 2004;233(3):729-39. | Cost-effectiveness study |
| Geller DA, Tsung A, Marsh JW, Dvorchik I, Gamblin TC, Carr BI. Outcome of 1000 liver cancer patients evaluated at the UPMC Liver Cancer Center. J Gastrointest Surg. 2006;10(1):63-8. | Wrong comparison |
| Geyik S, Akhan O, Abbasoglu O, Akinci D, Ozkan OS, Hamaloglu E, et al. Radiofrequency ablation of unresectable hepatic tumors. Diagn Interv Radiol. 2006;12(4):195-200. | Single cohort, no comparison |
| Gillams AR, Lees WR. Five-year survival following radiofrequency ablation of small, solitary, hepatic colorectal metastases. J Vasc Interv Radiol. 2008;19(5):712-7. | Single cohort, no comparison |
| Gillams AR, Lees WR. Five-year survival in 309 patients with colorectal liver metastases treated with radiofrequency ablation. Eur Radiol. 2009;19(5):1206-13. | Single cohort, no comparison |
| Gillams AR, Lees WR. Radio-frequency ablation of colorectal liver metastases in 167 patients. Eur Radiol. 2004;14(12):2261-7. | Single cohort, no comparison |
| Gillams AR, Lees WR. Radiofrequency ablation of colorectal liver metastases. Abdom Imaging. 2005;30(4):419-26. | Single cohort, no comparison |
| Gillams AR. Liver ablation therapy. Br J Radiol. 2004;77(921):713-23. | Single cohort, no comparison |
| Giorgio A, Tarantino L, de Stefano G, Coppola C, Ferraioli G. Complications after percutaneous saline-enhanced radiofrequency ablation of liver tumors: 3-year experience with 336 patients at a single center. AJR Am J Roentgenol. 2005;American Journal of Roentgenology. 184(1):207-11. | Single cohort, no comparison |
| Goldberg SN, Gazelle GS, Compton CC, Mueller PR, Tanabe KK. Treatment of intrahepatic malignancy with radiofrequency ablation: radiologic-pathologic correlation. Cancer. 2000;88(11):2452-63. | Single cohort, no comparison |
| Gomez Senent S, Gomez Raposo C, Mancenido Marcos N, Martin Chavarri S, Carrion Alonso G, Olveira Martin A, et al. Radiofrequency ablation for hepatocellular carcinoma and liver metastases: experience in Hospital La Paz. Clin Transl Oncol. 2006;8(9):688-91. | Single cohort, no comparison |
| Govaert K.M, Van Kessel C.S, Lolkema M, Ruers T.J.M, Rinkes I.H.M.B. Does radiofrequency ablation add to chemotherapy for unresectable liver metastases? Curr. Colorectal Cancer Rep. 2012;8(2):130-7. | Narrative review |
| Gravante G, Ong SL, Metcalfe MS, Strickland A, Dennison AR, Lloyd DM. Hepatic microwave ablation: a review of the histological changes following thermal damage. Liver Int. 2008;28(7):911-21. | Wrong outcomes |
| Gravante G, Overton J, Sorge R, Bhardwaj N, Metcalfe MS, Lloyd DM, et al. Radiofrequency ablation versus resection for liver tumours: an evidence-based approach to retrospective comparative studies. J Gastrointest Surg. 2011;15(2):378-87. | No quality appraisal of included studies |
| Greve JW. Alternative techniques for the treatment of colon carcinoma metastases in the liver: current status in The Netherlands. Scandinavian Journal of Gastroenterology Supplement. 2001;234(234):77-81. | Narrative review |
| Gugerbauer J, Warmuth M. Radiofrequency ablation for hepatocellular carcinoma and colorectal liver metastases (Structured abstract). Health Technology Assessment Database. 2011(2). | German |
| Gwak JH, Oh B-Y, Lee RA, Chung SS, Kim KH. Clinical applications of radio-frequency ablation in liver metastasis of colorectal cancer. J Korean Soc Coloproctology. 2011;27(4):202-10. | Single cohort, no comparison |
| Hager ED, Dziambor H, Hohmann D, Gallenbeck D, Stephan M, Popa C. Deep hyperthermia with radiofrequencies in patients with liver metastases from colorectal cancer. Anticancer Res. 1999;19(4C):3403-8. | Single cohort, no comparison |
| Hamada A, Yamakado K, Nakatsuka A, Uraki J, Kashima M, Takaki H, et al. Radiofrequency ablation for colorectal liver metastases: prognostic factors in non-surgical candidates. Jpn J Radiol. 2012;30(7):567-74. | Single cohort, no comparison |
| Hammill CW, Billingsley KG, Cassera MA, Wolf RF, Ujiki MB, Hansen PD. Outcome after laparoscopic radiofrequency ablation of technically resectable colorectal liver metastases. Ann Surg Oncol. 2011;18(7):1947-54. | Single cohort, no comparison |
| Han Y, Yan D, Xu F, Li X, Cai J. radiofrequency ablation versus liver resection for colorectal cancer liver metastasis: an updated systematic review and meta-analysis. | Systematic review without quality appraisal |
| He N, Jin Q, Wang D, Yang Y, et al. radiofrequency ablation vs. hepatic resection for resectable colorectal liver metastasis. 2016;36(4):514-18 | No matching or multivariate analysis |
| Health Quality O. Radio frequency ablation for primary liver cancer: an evidence-based analysis. Ontario Health Technology Assessment Series. 2004;4(8):1-50. | Primary liver cancer |
| Hildebrand P, Kleemann M, Roblick U, Mirow L, Birth M, Bruch H-P. Laparoscopic radiofrequency ablation of unresectable hepatic malignancies: indication, limitation and results. Hepatogastroenterology. 2007;54(79):2069-72. | Single cohort, no comparison |
| Hildebrand P, Kleemann M, Roblick UJ, Mirow L, Birth M, Leibecke T, et al. Radiofrequency-ablation of unresectable primary and secondary liver tumors: results in 88 patients. Langenbecks Arch Surg. 2006;391(2):118-23. | Wrong comparison |
| Hildebrand P, Leibecke T, Kleemann M, Mirow L, Birth M, Bruch H.P, et al. Influence of operator experience in radiofrequency ablation of malignant liver tumours on treatment outcome. Eur. J. Surg. Oncol. 2006;32(4):430-4. | Wrong comparison |
| Hofer S, Oberholzer C, Beck S, Looser C, Ludwig C. Ultrasound-guided radiofrequency ablation (RFA) for inoperable gastrointestinal liver metastases. Ultraschall Med. 2008;29(4):388-92. | Single cohort, no comparison |
| Hoffman AL, Wu SS, Obaid AK, French SW, Lois J, McMonigle M, et al. Histologic evaluation and treatment outcome after sequential radiofrequency ablation and hepatic resection for primary and metastatic tumors. Am Surg. 2002;68(12):1038-43. | Single cohort, no comparison |
| Hompes D, Prevoo W, Ruers T. Radiofrequency ablation as a treatment tool for liver metastases of colorectal origin. Cancer Imaging. 2011;11:23-30. | Narrative review |
| Hompes R, Fieuws S, Aerts R, Thijs M, Penninckx F, Topal B. Results of single-probe microwave ablation of metastatic liver cancer. Eur J Surg Oncol. 2010;36(8):725-30. | No separate results for CRLM |
| Howard JH, Tzeng C-WD, Smith JK, Eckhoff DE, Bynon JS, Wang T, et al. Radiofrequency ablation for unresectable tumors of the liver. Am Surg. 2008;74(7):594-600; discussion -1. | Single cohort, no comparison |
| Hubert C, Gras J, Goffette P, Grajeda JM, Van Beers BE, Laurence A, et al. Percutaneous and surgical radiofrequency ablation of liver malignancies: a single institutional experience. Acta Gastroenterol Belg. 2007;70(2):188-94. | Wrong comparison |
| Iannitti DA, Dupuy DE, Mayo-Smith WW, Murphy B. Hepatic radiofrequency ablation. Arch Surg. 2002;137(4):422-6; discussion 7. | Single cohort, no comparison |
| Ierardi AM, Floridi C, Fontana F, Chini C, Giorlando F, Piacentino F, et al. Microwave ablation of liver metastases to overcome the limitations of radiofrequency ablation. Radiol Med (Torino). 2013;118(6):949-61. | No separate results for CRLM |
| Izzo F. Other thermal ablation techniques: microwave and interstitial laser ablation of liver tumors. Ann Surg Oncol. 2003;10(5):491-7. | Narrative review |
| Jagad RB, Koshariya M, Kawamoto J, Papastratis P, Kefalourous H, Patris V, et al. Laparoscopic microwave ablation of liver tumors: our experience. Hepatogastroenterology. 2008;55(81):27-32. | No separate results for CRLM |
| Jakobs TF, Hoffmann RT, Trumm C, Reiser MF, Helmberger TK. Radiofrequency ablation of colorectal liver metastases: mid-term results in 68 patients. Anticancer Res. 2006;26(1B):671-80. | Single cohort, no comparison |
| Jaskolka JD, Asch MR, Kachura JR, Ho CS, Ossip M, Wong F, et al. Needle tract seeding after radiofrequency ablation of hepatic tumors. J Vasc Interv Radiol. 2005;16(4):485-91. | Case report |
| Jiang H-C, Liu L-X, Piao D-X, Xu J, Zheng M, Zhu A-L, et al. Clinical short-term results of radiofrequency ablation in liver cancers. World J Gastroenterol. 2002;8(4):624-30. | Wrong comparison |
| Jiao D-C, Zhou Q, Han X-W, Wang Y-F, Wu G, Ren J-Z, et al. Microwave ablation treatment of liver cancer with a 2,450-MHz cooled-shaft antenna: pilot study on safety and efficacy. Asian Pacific Journal of Cancer Prevention: Apjcp. 2012;13(2):737-42. | No separate results for CRLM |
| Jiao LR, Hansen PD, Havlik R, Mitry RR, Pignatelli M, Habib N. Clinical short-term results of radiofrequency ablation in primary and secondary liver tumors. Am J Surg. 1999;177(4):303-6. | Wrong comparison |
| Jones C, Badger SA, Ellis G. The role of microwave ablation in the management of hepatic colorectal metastases. Surg. 2011;9(1):33-7. | No quality appraisal of included studies |
| Joosten J, Jager G, Oyen W, Wobbes T, Ruers T. Cryosurgery and radiofrequency ablation for unresectable colorectal liver metastases. Eur J Surg Oncol. 2005;31(10):1152-9. | Wrong comparison |
| Kanellos I, Demetriades H, Blouhos K, Tsachalis T, Pramateftakis MG, Betsis D. Radio-frequency ablation of hepatic metastases from colorectal cancer. Techniques in Coloproctology. 2004;8(1). | 2 cases |
| Kang T.W, Lee M.W, Hye M.J, Song K.D, Lim S, Rhim H, et al. Percutaneous radiofrequency ablation of hepatic tumours: Factors affecting technical failure of artificial ascites formation using an angiosheath. Clin. Radiol. 2014;69(12):1249-58. | Wrong outcomes |
| Karanicolas PJ, Jarnagin WR, Gonen M, Tuorto S, Allen PJ, DeMatteo RP, et al. Long-term outcomes following tumor ablation for treatment of bilateral colorectal liver metastases. JAMA Surg. 2013;148(7):597-601. | No matching or multivariate analysis |
| Kele PG, de Jong KP, van der Jagt EJ. Increase in volume of ablation zones during follow-up is highly suggestive of ablation site recurrence in colorectal liver metastases treated with radiofrequency ablation. J Vasc Interv Radiol. 2012;23(4):537-44. | Single cohort, no comparison |
| Kennedy TJ, Cassera MA, Khajanchee YS, Diwan TS, Hammill CW, Hansen PD. Laparoscopic radiofrequency ablation for the management of colorectal liver metastases: 10-year experience. J Surg Oncol. 2013;107(4):324-8. | Single cohort, no comparison |
| Khajanchee YS, Hammill CW, Cassera MA, Wolf RF, Hansen PD. Hepatic resection vs minimally invasive radiofrequency ablation for the treatment of colorectal liver metastases: a Markov analysis. Arch Surg. 2011;146(12):1416-23. | Markov model |
| Kim WW, Kim KH, Kim SH, Kim JS, et al. Comparison of hepatic resection and radiofrequency ablation for the treatment of colorectal liver metastasis. Indian J Surg. 2015;77(3):1126-30. | No matching or multivariate analysis |
| Kingham TP, Tanoue M, Eaton A, Rocha FG, Do R, Allen P, et al. Patterns of recurrence after ablation of colorectal cancer liver metastases. Ann Surg Oncol. 2012;19(3):834-41. | Single cohort, no comparison |
| Knudsen AR, Kannerup A-S, Mortensen FV, Nielsen DT. Radiofrequency ablation of colorectal liver metastases downstaged by chemotherapy. Acta Radiol. 2009;50(7):716-21. | No full-text |
| Ko S, Jo H, Yun S, Park E, Kim S, Seo H-I. Comparative analysis of radiofrequency ablation and resection for resectable colorectal liver metastases. World J Gastroenterol. 2014;20(2):525-31. | No matching or multivariate analysis |
| Kornprat P, Jarnagin WR, DeMatteo RP, Fong Y, Blumgart LH, D'Angelica M. Role of intraoperative thermoablation combined with resection in the treatment of hepatic metastasis from colorectal cancer. Arch Surg. 2007;142(11):1087-92. | Single cohort, no comparison |
| Kulikovsky V.F, Oleynik N.V, Soloshenko A.V, Naumov A.V, Storojilov D.A. Surgical management of colorectal cancer liver metastases. Res. J. Pharm., Biol. Chem. Sci. 2014;5(5):1108-11. | No matching or multivariate analysis |
| Kulkarni S, Shetty NS, Polnaya AM, Patil S, Gala K, et al. Early outcomes of radiofrequency ablation in unresectable metastatic colorectal cancer from a tertiary cancer hospital in India. Indian J Radiol Imaging. 2017;27(2):200-206. | Single cohort, no comparison |
| Kuvshinoff BW, Ota DM. Radiofrequency ablation of liver tumors: influence of technique and tumor size. Surgery. 2002;132(4):605-11; discussion 11-2. | Single cohort, no comparison |
| Kwan BYM, Kielar AZ, El-Maraghi RH, Garcia LM. Retrospective review of efficacy of radiofrequency ablation for treatment of colorectal cancer liver metastases from a Canadian perspective. Can Assoc Radiol J. 2014;65(1):77-85. | Wrong comparison |
| Labori KJ, Schulz A, drolsum A, Gronlie Guren M, et al. Radiofrequency ablation of unresectable colorectal liver metastases: trends in management and outcome during a decade at a single center. Acta Radiologica Open. 2015;4(7):1-9. | Single cohort, no comparison |
| Lahat E, Eshkenazy R, Zendel A, Zakai BB, Maor M, Dreznik Y, et al. Complications after percutaneous ablation of liver tumors: a systematic review. Hepatobiliary surg. 2014;3(5):317-23. | No quality appraisal of included studies, no separate results for CRLM |
| Lao OB, Farjah F, Flum DR, Yeung RS. Adverse events after radiofrequency ablation of unresectable liver tumors: a single-center experience. Am J Surg. 2009;198(1):76-82. | Single cohort, no comparison |
| Lau TN, Lo RHG, Tan BS. Colorectal hepatic metastases: Role of radiofrequency ablation. Ann Acad Med Singapore. 2003;32(2):212-8. | Narrative review |
| Leblanc F, Fonck M, Brunet R, Becouarn Y, Mathoulin-Pelissier S, Evrard S. Comparison of hepatic recurrences after resection or intraoperative radiofrequency ablation indicated by size and topographical characteristics of the metastases. Eur J Surg Oncol. 2008;34(2):185-90. | Single cohort, no comparison |
| Leblanc F, Fonck M, Brunet R, Becouarn Y, Mathoulin-Pelissier S, Evrard S. Comparison of hepatic recurrences after resection or intraoperative radiofrequency ablation indicated by size and topographical characteristics of the metastases. Eur. J. Surg. Oncol. 2008;34(2):185-90. | Double |
| Lencioni R, Goletti O, Armillotta N, Paolicchi A, Moretti M, Cioni D, et al. Radio-frequency thermal ablation of liver metastases with a cooled-tip electrode needle: results of a pilot clinical trial. European Radiology. 1998;8(7):1205-11. | Single cohort, no comparison |
| Lermite E, Lebigot J, Oberti F, Pessaux P, Aube C, Cales P, et al. Radiofrequency thermal ablation of liver carcinoma. Prospective study of 82 lesions. Gastroenterol Clin Biol. 2006;30(1):130-5. | Single cohort, no comparison |
| Leung EYL, Roxburgh CSD, Leen E, Horgan PG. Combined resection and radiofrequency ablation for bilobar colorectal cancer liver metastases. Hepatogastroenterology. 2010;57(97):41-6. | No matching or multivariate analysis |
| Liang P, Dong B, Yu X, Yang Y, Yu D, Su L, et al. Prognostic factors for percutaneous microwave coagulation therapy of hepatic metastases. AJR Am J Roentgenol. 2003;American Journal of Roentgenology. 181(5):1319-25. | No separate results for CRLM |
| Liang P, Wang Y, Yu X, Dong B. Malignant liver tumors: treatment with percutaneous microwave ablation--complications among cohort of 1136 patients. Radiology. 2009;251(3):933-40. | No separate results for CRLM |
| Liu C-H, Arellano RS, Uppot RN, Samir AE, Gervais DA, Mueller PR. Radiofrequency ablation of hepatic tumours: effect of post-ablation margin on local tumour progression. Eur Radiol. 2010;20(4):877-85. | Single cohort, no comparison |
| Liu C-H, Yu C-Y, Chang W-C, Dai M-S, Hsiao C-W, Chou Y-C. Radiofrequency ablation of hepatic metastases: factors influencing local tumor progression. Ann Surg Oncol. 2014;21(9):3090-5. | Single cohort, no comparison |
| Liu Y, Li S, Wan X, Li Y, Li B, Zhang Y, et al. Efficacy and safety of thermal ablation in patients with liver metastases. Eur J Gastroenterol Hepatol. 2013;25(4):442-6. | No separate results for CRLM |
| Livraghi T, Meloni F, Solbiati L, Zanus G, Collaborative Italian Group using As. Complications of microwave ablation for liver tumors: results of a multicenter study. Cardiovasc Intervent Radiol. 2012;35(4):868-74. | No separate results for CRLM |
| Livraghi T, Solbiati L, Meloni F, Ierace T, Goldberg SN, Gazelle GS. Percutaneous radiofrequency ablation of liver metastases in potential candidates for resection: the "test-of-time approach". Cancer. 2003;97(12):3027-35. | Single cohort, no comparison |
| Livraghi T, Solbiati L, Meloni MF, Gazelle GS, Halpern EF, Goldberg SN. Treatment of focal liver tumors with percutaneous radio-frequency ablation: complications encountered in a multicenter study. Radiology. 2003;226(2):441-51. | No separate results for CRLM |
| Lorentzen T, Skjoldbye BO, Nolsoe CP. Microwave ablation of liver metastases guided by contrast-enhanced ultrasound: experience with 125 metastases in 39 patients. Ultraschall Med. 2011;32(5):492-6. | No separate results for MWA alone |
| Low SCS, Lo RHG, Lau T-N, Ooi LLPJ, Ho C-K, Tan B-S, et al. Image-guided radiofrequency ablation of liver malignancies: experience at Singapore General Hospital. Ann Acad Med Singapore. 2006;35(12):851-7. | Wrong comparison |
| Lubner MG, Brace CL, Ziemlewicz TJ, Hinshaw JL, Lee FT, Jr. Microwave ablation of hepatic malignancy. SEMIN. 2013;30(1):56-66. | Narrative review |
| Machi J, Oishi AJ, Sumida K, Sakamoto K, Furumoto NL, Oishi RH, et al. Long-term outcome of radiofrequency ablation for unresectable liver metastases from colorectal cancer: evaluation of prognostic factors and effectiveness in first- and second-line management. Cancer J. 2006;12(4):318-26. | Single cohort, no comparison |
| Machi J, Uchida S, Sumida K, Limm WM, Hundahl SA, Oishi AJ, et al. Ultrasound-guided radiofrequency thermal ablation of liver tumors: percutaneous, laparoscopic, and open surgical approaches. J Gastrointest Surg. 2001;5(5):477-89. | Single cohort, no comparison |
| Majerovic M, Augustin G, Jelincic Z, Bukovic D, Kekez T, Matosevic P, et al. Radiofrequency ablation as locoregional therapy for unresectable hepatic malignancies: initial results in 24 patients with 5-years follow-up. Coll Antropol. 2008;32(3):703-7. | Single cohort, no comparison |
| Martin RCG, 2nd, Scoggins CR, McMasters KM. A phase II study of radiofrequency ablation of unresectable metastatic colorectal cancer with hepatic arterial infusion pump chemotherapy. J Surg Oncol. 2006;93(5):387-93. | Wrong intervention: RFA + chemotherapy |
| Martin RCG, Scoggins CR, McMasters KM. Microwave hepatic ablation: initial experience of safety and efficacy. J Surg Oncol. 2007;96(6):481-6. | <10 pts with CRLM |
| Mazziotti A, Grazi GL, Gardini A, Cescon M, Pierangeli F, Ercolani G, et al. An appraisal of percutaneous treatment of liver metastases. Liver Transpl Surg. 1998;4(4):271-5. | Single cohort, <10 pts with CRLM |
| McKay A, Dixon E, Taylor M. Current role of radiofrequency ablation for the treatment of colorectal liver metastases. Br J Surg. 2006;93(10):1192-201. | Only Medline, no quality appraisal of included studies |
| McKay A, Kutnikoff T, Taylor M. A cost-utility analysis of treatments for malignant liver tumours: a pilot project. Hpb. 2007;9(1):42-51. | Cost-effectiveness study |
| Mima K, Beppu T, Chikamoto A, Miyamoto Y, Nakagawa S, Kuroki H, et al. Hepatic resection combined with radiofrequency ablation for initially unresectable colorectal liver metastases after effective chemotherapy is a safe procedure with a low incidence of local recurrence. Int J Clin Oncol. 2013;18(5):847-55. | No matching or multivariate analysis |
| Minami Y, Kudo M, Chung H, Inoue T, Takahashi S, Hatanaka K, et al. Percutaneous radiofrequency ablation of sonographically unidentifiable liver tumors. Feasibility and usefulness of a novel guiding technique with an integrated system of computed tomography and sonographic images. Oncology. 2007;1:111-6. | Single cohort, <10 pts with CRLM |
| Minami Y, Kudo M. Radiofrequency ablation of liver metastases from colorectal cancer: a literature review. Gut and liver. 2013;7(1):1-6. | Narrative review |
| Morgan JH, 3rd, Royer GM, Hackett P, Gamblin TC, McCampbell BL, Conforti A, et al. Radio-frequency ablation of large, nonresectable hepatic tumors. Am Surg. 2004;70(12):1035-8. | Wrong comparison |
| Morikawa S, Inubushi T, Kurumi Y, Naka S, Sato K, Tani T, et al. MR-guided microwave thermocoagulation therapy of liver tumors: initial clinical experiences using a 0.5 T open MR system. J Magn Reson Imaging. 2002;16(5):576-83. | No separate results for CRLM |
| Mulier S, Mulier P, Ni Y, Miao Y, Dupas B, Marchal G, et al. Complications of radiofrequency coagulation of liver tumours. Br J Surg. 2002;89(10):1206-22. | No quality appraisal of included studies |
| Mulier S, Ni Y, Jamart J, Michel L, Marchal G, Ruers T. Radiofrequency ablation versus resection for resectable colorectal liver metastases: time for a randomized trial? Ann Surg Oncol. 2008;15(1):144-57. | No quality appraisal of included studies |
| Mulier S, Ruers T, Jamart J, Michel L, Marchal G, Ni Y. Radiofrequency ablation versus resection for resectable colorectal liver metastases: time for a randomized trial? An update. Digestive Surgery. 2008;25(6):445-60. | Same review as Mulier 2008 |
| Nagata Y, Hiraoka M, Akuta K, Abe M, Takahashi M, Jo S, et al. Radiofrequency thermotherapy for malignant liver tumors. Cancer. 1990;65(8):1730-6. | Single cohort, no comparison |
| Nagata Y, Hiraoka M, Nishimura Y, Masunaga S, Mitumori M, Okuno Y, et al. Clinical results of radiofrequency hyperthermia for malignant liver tumors. Int J Radiat Oncol Biol Phys. 1997;38(2):359-65. | Single cohort, no comparison |
| Navarra G, Ayav A, Weber JC, Jensen SL, Smadga C, Nicholls JP, et al. Short- and-long term results of intraoperative radiofrequency ablation of liver metastases. Int J Colorectal Dis. 2005;20(6):521-8. | Single cohort, no comparison |
| Neophytou C, Valachis A, Bhalla A. A meta-analysis of radiofrequency ablation for colorectal liver metastases. Colorectal Dis. 2014;16:79-80. | Poster |
| Ng KK-C, Lam C-M, Poon RT-P, Ai V, Tso W-K, Fan S-T. Thermal ablative therapy for malignant liver tumors: a critical appraisal. J Gastroenterol Hepatol. 2003;18(6):616-29. | Narrative review |
| Nicholl M.B, Conway W.C, Ye X, Bilchik A, Singh G. Should microwave energy be preferred to radiofrequency energy for ablation of malignant liver tumors? J. Intervent. Oncol. 2010;3(1):12-6. | No full-text |
| Nielsen K, van Tilborg AAJM, Meijerink MR, Macintosh MO, Zonderhuis BM, de Lange ESM, et al. Incidence and treatment of local site recurrences following RFA of colorectal liver metastases. World J Surg. 2013;37(6):1340-7. | Single cohort, no comparison |
| North DA, Groeschl RT, Sindram D, Martinie JB, Iannitti DA, Bloomston M, et al. Microwave ablation for hepatic malignancies: a call for standard reporting and outcomes. Am J Surg. 2014;208(2):284-94. | No separate results for CRLM |
| Nouso K, Kobayashi Y, Nakamura S, Uematsu S, Shiraga K, Iwadou S, et al. Application of radiofrequency ablation for the treatment of metastatic liver cancers. Hepatogastroenterology. 2010;57(97):117-20. | Single cohort, no comparison |
| Ogata Y, Uchida S, Hisaka T, Horiuchi H, Mori S, Ishibashi N, et al. Intraoperative thermal ablation therapy for small colorectal metastases to the liver. Hepatogastroenterology. 2008;55(82-83):550-6. | Single cohort, no comparison |
| Ong SL, Gravante G, Metcalfe MS, Strickland AD, Dennison AR, Lloyd DM. Efficacy and safety of microwave ablation for primary and secondary liver malignancies: a systematic review. Eur J Gastroenterol Hepatol. 2009;21(6):599-605. | Only Medline, no quality appraisal of included studies |
| Oshowo A, Gillams A, Harrison E, Lees WR, Taylor I. Comparison of resection and radiofrequency ablation for treatment of solitary colorectal liver metastases.N Br J Surg. 2003;90(10):1240-3. | No matching or multivariate analysis |
| Oshowo A, Gillams AR, Lees WR, Taylor I. Radiofrequency ablation extends the scope of surgery in colorectal liver metastases. Eur J Surg Oncol. 2003;29(3):244-7. | Wrong comparison |
| Panpikoon T, Treesit T, Thapaneeyakorn J, Wedsart B, Inman T. Efficacy of percutaneous radiofrequency ablation of hepatic malignant tumors using a perfused-cooled electrode. J Med Assoc Thai. 2013;96(1):77-82. | Single cohort, <10 pts with CRLM |
| Park MJ, Kim TH, Lee KM, Cheong JY, Kim JK. Radiofrequency ablation of metastatic liver masses: recurrence patterns and prognostic factors based on radiologic features. Hepatogastroenterology. 2013;60(123):563-7. | Single cohort, no comparison |
| Pawlik TM, Izzo F, Cohen DS, Morris JS, Curley SA. Combined resection and radiofrequency ablation for advanced hepatic malignancies: results in 172 patients. Ann Surg Oncol. 2003;10(9):1059-69. | Single cohort, no comparison |
| Pepple P.T, Gerber D.A. Laparoscopic-assisted ablation of hepatic tumors: A review. Semin. Intervent. Radiol. 2014;31(2):125-8. | Narrative review |
| Petrovich Z, Langholz B, Astrahan M, Emami B. Deep microwave hyperthermia for metastatic tumors of the liver. Recent Results in Cancer Research. 1988;107:244-8. | No separate results for CRLM |
| Poggi G, Riccardi A, Quaretti P, Teragni C, Delmonte A, Amatu A, et al. Complications of percutaneous radiofrequency thermal ablation of primary and secondary lesions of the liver. Anticancer Res. 2007;27(4C):2911-6. | No separate results for CRLM |
| Polignano FM, Quyn AJ, Sanjay P, Henderson NA, Tait IS. Totally laparoscopic strategies for the management of colorectal cancer with synchronous liver metastasis. Surg Endosc. 2012;26(9):2571-8. | Single cohort, no comparison |
| Poon RT, Ng KK, Lam CM, Ai V, Yuen J, Fan ST, et al. Learning curve for radiofrequency ablation of liver tumors: prospective analysis of initial 100 patients in a tertiary institution. Ann Surg. 2004;239(4):441-9. | Wrong comparison |
| Popescu I, Alexandrescu S, Croitoru A, Boros M. Strategies to convert to resectability the initially unresectable colorectal liver metastases. Hepatogastroenterology. 2009;56(91-92):739-44. | No matching or multivariate analysis |
| Poulou LS, Thanos L, Ziakas PD, Merikas E, Achimastos A, et al. thermal ablation may improve outcomes in patients with colorectal liver metastasis: a case-control study. 2017;22(3):673-78. | Wrong comparison |
| Poulou LS, Ziakas PD, Xila V, Vakrinos G, Malagari K, Syrigos KN, et al. Percutaneous radiofrequency ablation for unresectable colorectal liver metastases: time for shadows to disperse. Rev Recent Clin Trials. 2009;4(3):140-6. | Only Medline, no quality appraisal of included studies |
| Qiu J, Chen S, Wu H. Long-term outcomes after hepatic resection combined with radiofrequency ablation for initially unresectable multiple and bilobar liver malignancies. J Surg Res. 2014;188(1):14-20. | Single cohort, no comparison |
| Radiofrequency ablation of unresectable hepatic tumors. Tecnologica MAP Supplement. 2000. | No methodological information |
| Rasmussen F. Radiofrequency ablation of liver metastases improves the survival rate of patients with metastatic colorectal disease. Acta Radiol. 2007;48(3):250-1. | Editorial |
| Rath GK, Julka PK, Thulkar S, Sharma DN, Bahl A, Bhatnagar S. Radiofrequency ablation of hepatic metastasis: results of treatment in forty patients. J Cancer Res Ther. 2008;4(1):14-7. | Single cohort, no comparison |
| Razafindratsira T, Isambert M, Evrard S. Complications of intraoperative radiofrequency ablation of liver metastases. HPB. 2011;13(1):15-23. | Only Medline, no quality appraisal of included studies |
| Reuter NP, Woodall CE, Scoggins CR, McMasters KM, Martin RCG. Radiofrequency ablation vs. resection for hepatic colorectal metastasis: therapeutically equivalent? J Gastrointest Surg. 2009;13(3):486-91. | No matching or multivariate analysis |
| Rhim H, Lim HK, Kim Y-s, Choi D, Lee WJ. Radiofrequency ablation of hepatic tumors: lessons learned from 3000 procedures. J Gastroenterol Hepatol. 2008;23(10):1492-500. | Single cohort, no comparison |
| Ribeiro MAF, Jr., Rodrigues JJG, Habr-Gama A, Chaib E, D'Ipolitto G, Fonseca AZ, et al. Radiofrequency ablation of primary and metastatic liver tumors--4 years experience. Hepatogastroenterology. 2007;54(76):1170-5. | Wrong comparison |
| Ritz J-P, Lehmann KS, Reissfelder C, Albrecht T, Frericks B, Zurbuchen U, et al. Bipolar radiofrequency ablation of liver metastases during laparotomy. First clinical experiences with a new multipolar ablation concept. Int J Colorectal Dis. 2006;21(1):25-32. | Single cohort, no comparison |
| Rocha FG, D'Angelica M. Treatment of liver colorectal metastases: role of laparoscopy, radiofrequency ablation, and microwave coagulation. J Surg Oncol. 2010;102(8):968-74. | Narrative review |
| Rose DM, Allegra DP, Bostick PJ, Foshag LJ, Bilchik AJ. Radiofrequency ablation: a novel primary and adjunctive ablative technique for hepatic malignancies. Am Surg. 1999;65(11):1009-14. | Single cohort, no comparison |
| Rossi S, Di Stasi M, Buscarini E, Quaretti P, Garbagnati F, Squassante L, et al. Percutaneous RF interstitial thermal ablation in the treatment of hepatic cancer. AJR Am J Roentgenol. 1996;American Journal of Roentgenology. 167(3):759-68. | Single cohort, no comparison |
| Ruers TJM, Joosten JJ, Wiering B, Langenhoff BS, Dekker HM, Wobbes T, et al. Comparison between local ablative therapy and chemotherapy for non-resectable colorectal liver metastases: a prospective study. Ann Surg Oncol. 2007;14(3):1161-9. | No matching or multivariate analysis |
| Sainani N.I, Shyn P.B, Tatli S, Morrison P.R, Tuncali K, Silverman S.G. PET/CT-guided radiofrequency and cryoablation: Is tumor fluorine-18 fluorodeoxyglucose activity dissipated by thermal ablation? J. Vasc. Intervent. Radiol. 2011;22(3):354-60. | Only 2 pts with CRLM |
| Scaife CL, Curley SA, Izzo F, Marra P, Delrio P, Daniele B, et al. Feasibility of adjuvant hepatic arterial infusion of chemotherapy after radiofrequency ablation with or without resection in patients with hepatic metastases from colorectal cancer. Ann Surg Oncol. 2003;10(4):348-54. | Wrong intervention |
| Schumacher G, Eisele R, Spinelli A, Schmidt SC, Jacob D, Pratschke J, et al. Indications for hand-assisted laparoscopic radiofrequency ablation for liver tumors. J Laparoendosc Adv Surg Tech A. 2007;Part A. 17(2):153-9. | Single cohort, no comparison |
| Scudamore CH, Lee SI, Patterson EJ, Buczkowski AK, July LV, Chung SW, et al. Radiofrequency ablation followed by resection of malignant liver tumors. Am J Surg. 1999;177(5):411-7. | Single cohort, no comparison |
| Seidenfeld J, Korn A, Aronson N. Radiofrequency ablation of unresectable liver metastases. J Am Coll Surg. 2002;195(3):378-86. | Only Medline, no quality appraisal of included studies |
| Senent S.G, Raposo C.G, Marcos N.M, Chavarri S.M, Alonso G.C, Martin A.O, et al. Radiofrequency ablation for hepatocellular carcinoma and liver metastases: Experience in Hospital La Paz. Clin. Transl. Oncol. 2006;8(9):688-91. | Double |
| Seror O, Haddar D, N'Kontchou G, Ajavon Y, Trinchet J-C, Beaugrand M, et al. Radiofrequency ablation for the treatment of liver tumors in the caudate lobe. J Vasc Interv Radiol. 2005;16(7):981-90. | Wrong comparison, only 2 pts with CRLM |
| Sgouros J, Cast J, Garadi KK, Belechri M, Breen DJ, Monson JR, et al. Chemotherapy plus percutaneous radiofrequency ablation in patients with inoperable colorectal liver metastases. World J Gastrointest Oncol. 2011;3(4):60-6. | Wrong comparison |
| Shen P, Geisinger KR, Zagoria R, Levine EA. Pathologic correlation study of microwave coagulation therapy for hepatic malignancies using a three-ring probe. J Gastrointest Surg. 2007;11(5):603-11. | <10 pts with CRLM |
| Shetty SK, Rosen MP, Raptopoulos V, Goldberg SN. Cost-effectiveness of percutaneous radiofrequency ablation for malignant hepatic neoplasms. J Vasc Interv Radiol. 2001;12(7):823-33. | Cost study |
| Shibata T, Murakami T, Ogata N. Percutaneous microwave coagulation therapy for patients with primary and metastatic hepatic tumors during interruption of hepatic blood flow. Cancer. 2000;88(2):302-11. | No separate results for CRLM |
| Shimada S, Hirota M, Beppu T, Matsuda T, Hayashi N, Tashima S, et al. Complications and management of microwave coagulation therapy for primary and metastatic liver tumors. Surgery Today. 1998;28(11):1130-7. | No separate results for CRLM |
| Shukla PJ, Barreto SG. Surgery for malignant liver tumors. J Cancer Res Ther. 2009;5(3):154-60. | No quality appraisal of included studies |
| Siperstein A, Garland A, Engle K, Rogers S, Berber E, String A, et al. Laparoscopic radiofrequency ablation of primary and metastatic liver tumors. Technical considerations. Surg Endosc. 2000;14(4):400-5. | Single cohort, no comparison |
| Siperstein AE, Berber E, Ballem N, Parikh RT. Survival after radiofrequency ablation of colorectal liver metastases: 10-year experience. Ann Surg. 2007;246(4):559-65; discussion 65-7. | Single cohort, no comparison |
| Snoeren N, Nijkamp MW, Berendsen T, Govaert KM, van Kessel CS, Borel Rinkes IHM, et al. Multipolar radiofrequency ablation for colorectal liver metastases close to major hepatic vessels. Surg. 2015;13(2):77-82. | Single cohort: all received RFA |
| Sofocleous CT, Petre EN, Gonen M, Brown KT, Solomon SB, Covey AM, et al. CT-guided radiofrequency ablation as a salvage treatment of colorectal cancer hepatic metastases developing after hepatectomy. J Vasc Interv Radiol. 2011;22(6):755-61. | Single cohort, no comparison |
| Solbiati L, Ahmed M, Cova L, Ierace T, Brioschi M, Goldberg SN. Small liver colorectal metastases treated with percutaneous radiofrequency ablation: local response rate and long-term survival with up to 10-year follow-up. Radiology. 2012;265(3):958-68. | Single cohort, no comparison |
| Solbiati L, Goldberg SN, Ierace T, Dellanoce M, Livraghi T, Gazelle GS. Radio-frequency ablation of hepatic metastases: postprocedural assessment with a US microbubble contrast agent--early experience. Radiology. 1999;211(3):643-9. | Single cohort, no comparison |
| Solbiati L, Goldberg SN, Ierace T, Livraghi T, Meloni F, Dellanoce M, et al. Hepatic metastases: percutaneous radio-frequency ablation with cooled-tip electrodes. Radiology. 1997;205(2):367-73. | Single cohort, no comparison |
| Solbiati L, Ierace T, Tonolini M, Osti V, Cova L. Radiofrequency thermal ablation of hepatic metastases. Eur J Ultrasound. 2001;13(2):149-58. | Single cohort, no comparison |
| Solbiati L, Livraghi T, Goldberg SN, Ierace T, Meloni F, Dellanoce M, et al. Percutaneous radio-frequency ablation of hepatic metastases from colorectal cancer: long-term results in 117 patients. Radiology. 2001;221(1):159-66. | Single cohort, no comparison |
| Sorensen SM, Mortensen FV, Nielsen DT. Radiofrequency ablation of colorectal liver metastases: long-term survival.[Reprint in Ugeskr Laeger. 2008 Apr 14;170(16):1361-3; PMID: 18433601]. Acta Radiol. 2007;48(3):253-8. | No full-text |
| Stang A, Fischbach R, Teichmann W, Bokemeyer C, Braumann D. A systematic review on the clinical benefit and role of radiofrequency ablation as treatment of colorectal liver metastases. Eur J Cancer. 2009;45(10):1748-56. | Only Medline, no quality appraisal of included studies |
| Stang A, Oldhafer KJ, Weilert H, Keles H, Donati M. Selection criteria for radiofrequency ablation for colorectal liver metastases in the era of effective systemic therapy: a clinical score based proposal. BMC Cancer. 2014;14(500). | Single cohort, no comparison |
| Stattner S, Primavesi F, Yip VS, Jones RP, Ofner D, Malik HZ, et al. Evolution of surgical microwave ablation for the treatment of colorectal cancer liver metastasis: review of the literature and a single centre experience. SURG. 2015;45(4):407-15. | No separate results for MWA alone |
| Stintzing S, Grothe A, Hendrich S, Hoffmann R-T, Heinemann V, Rentsch M, et al. Percutaneous radiofrequency ablation (RFA) or robotic radiosurgery (RRS) for salvage treatment of colorectal liver metastases. Acta Oncol. 2013;52(5):971-7. | Wrong comparison |
| Sun W. Advanced local therapies for the treatment of limited systemic mCRC. Oncology. 2014;28(11):1. | Commentary |
| Suppiah A, White TJ, Roy-Choudhury SH, Breen DJ, Cast J, Maraveyas A, et al. Long-term results of percutaneous radiofrequency ablation of unresectable colorectal hepatic metastases: final outcomes. Digestive Surgery. 2007;24(5):358-60. | Single cohort, no comparison |
| Takaki H, Yamakado K, Nakatsuka A, Yamada T, Shiraki K, Takei Y, et al. Frequency of and risk factors for complications after liver radiofrequency ablation under CT fluoroscopic guidance in 1500 sessions: single-center experience. AJR Am J Roentgenol. 2013;American Journal of Roentgenology. 200(3):658-64. | Single cohort, no comparison |
| Tanis E, Nordlinger B, Mauer M, Sorbye H, van Coevorden F, Gruenberger T, et al. Local recurrence rates after radiofrequency ablation or resection of colorectal liver metastases. Analysis of the European Organisation for Research and Treatment of Cancer #40004 and #40983. Eur J Cancer. 2014;50(5):912-9. | No matching or multivariate analysis |
| Tepel J, Hinz S, Klomp HJ, Kapischke M, Kremer B. Intraoperative radiofrequency ablation (RFA) for irresectable liver malignancies. Eur J Surg Oncol. 2004;30(5):551-5. | Single cohort, no comparison |
| Topal B, Hompes D, Aerts R, Fieuws S, Thijs M, Penninckx F. Morbidity and mortality of laparoscopic vs. open radiofrequency ablation for hepatic malignancies. Eur J Surg Oncol. 2007;33(5):603-7. | Wrong comparison: RFA vs. RFA |
| Tropea A, Biondi A, Corsaro A, Donati M, Basile F, Gruttadauria S. Combined microwave thermal ablation and liver resection for single step treatment of otherwise unresectable colorectal liver metastases; a monoistitutional experiences. Eur Rev Med Pharmacol Sci. 2014;18(2 Suppl):6-10. | Only 5 pts with MWA |
| Tsai S, Pawlik TM. Outcomes of ablation versus resection for colorectal liver metastases: are we comparing apples with oranges? Ann Surg Oncol. 2009;16(9):2422-8. | Letter |
| Ungureanu BS, Sandulescu L, Surlin V, Sparchez Z, Saftoiu A. Surgical hepatic resection vs. ultrasonographic guided radiofrequency ablation in colorectal liver metastases: what should we choose? Med. 2014;16(2):145-51. | Narrative review |
| Van den Eynde M, Hendlisz A. Treatment of colorectal liver metastases: a review. Rev Recent Clin Trials. 2009;4(1):56-62. | Narrative review |
| Van Tilborg AAJM, Meijerink MR, Sietses C, Van Waesberghe JHTM, Mackintosh MO, Meijer S, et al. Long-term results of radiofrequency ablation for unresectable colorectal liver metastases: a potentially curative intervention. Br J Radiol. 2011;84(1002):556-65. | Single cohort, no comparison |
| Vietti-Violi N, Bize PE, Duran R, Demartines N, et al. Metastasectomy or radiofrequency ablation for treatment for colorectal cancer liver metastasis: comparison of recurrence rates and identification of risk factors for local recurrence. J clin oncology. 2016;34(4):769. | Wrong outcomes |
| Veltri A, Gazzera C, Rotondella C, Camerano F, Busso M, Gandini G. Image-guided microwave ablation of hepatic tumours: preliminary experience. Radiol Med (Torino). 2012;117(3):378-92. | <10 pts with CRLM |
| Veltri A, Guarnieri T, Gazzera C, Busso M, Solitro F, Fora G, et al. Long-term outcome of radiofrequency thermal ablation (RFA) of liver metastases from colorectal cancer (CRC): size as the leading prognostic factor for survival. Radiol Med (Torino). 2012;117(7):1139-51. | Single cohort, no comparison |
| Veltri A, Sacchetto P, Tosetti I, Pagano E, Fava C, Gandini G. Radiofrequency ablation of colorectal liver metastases: small size favorably predicts technique effectiveness and survival. Cardiovasc Intervent Radiol. 2008;31(5):948-56. | Single cohort, no comparison |
| Vlad L, Osian G, Branda H, Sparchez Z, Miclaus D, Furcea L, et al. Radiofrequency by open surgical approach in the treatment of hepatic tumours: early experience with 14 cases. Rom J Gastroenterol. 2003;12(2):113-8. | Single cohort, no comparison |
| Vogl TJ, Farshid P, Naguib NNN, Darvishi A, Bazrafshan B, Mbalisike E, et al. Thermal ablation of liver metastases from colorectal cancer: radiofrequency, microwave and laser ablation therapies. Radiol Med (Torino). 2014;119(7):451-61. | Narrative review |
| von Breitenbuch P, Kohl G, Guba M, Geissler E, Jauch KW, Steinbauer M. Thermoablation of colorectal liver metastases promotes proliferation of residual intrahepatic neoplastic cells. Surgery. 2005;138(5):882-7. | Mice |
| Votrubova J, Horejs J, Peskova M, Svab J, Krska Z. Radiofrequency thermoablation of hepatic tumours. Ceska Radiol. 2002;56(3):145-50. | Czech |
| Wang X, Sofocleous CT, Erinjeri JP, Petre EN, Gonen M, Do KG, et al. Margin size is an independent predictor of local tumor progression after ablation of colon cancer liver metastases. Cardiovasc Intervent Radiol. 2013;36(1):166-75. | Single cohort, no comparison |
| Wei A.C, Kachura J.R. Radiofrequency ablation in the treatment of isolated liver metastases from colorectal cancer. Cochrane Database Syst. Rev. 2007(1). | Updated by Cirocchi |
| Weng M, Zhang Y, Zhou D, Yang Y, Tang Z, Zhao M, et al. Radiofrequency ablation versus resection for colorectal cancer liver metastases: a meta-analysis. PLoS ONE [Electronic Resource]. 2012;7(9):e45493. | No quality appraisal of included studies |
| Wertenbroek MWJLAE, Schepers M, Kamminga-Rasker HJ, Bottema JT, Muller Kobold AC, Roelofsen H, et al. Clinical outcome, proteome kinetics and angiogenic factors in serum after thermoablation of colorectal liver metastases. BMC Cancer. 2013;13(266). | No matching or multivariate analysis |
| White RR, Avital I, Sofocleous CT, Brown KT, Brody LA, Covey A, et al. Rates and patterns of recurrence for percutaneous radiofrequency ablation and open wedge resection for solitary colorectal liver metastasis. J Gastrointest Surg. 2007;11(3):256-63. | No matching or multivariate analysis |
| White TJ, Roy-Choudhury SH, Breen DJ, Cast J, Maraveyas A, Smyth EF, et al. Percutaneous radiofrequency ablation of colorectal hepatic metastases - initial experience. An adjunct technique to systemic chemotherapy for those with inoperable colorectal hepatic metastases. Digestive Surgery. 2004;21(4):314-20. | Wrong comparison |
| Wong J, Lee KF, Lee PSF, Ho SSM, Yu SCH, Ng WWC, et al. Radiofrequency ablation for 110 malignant liver tumours: preliminary results on percutaneous and surgical approaches. ASIAN J. 2009;32(1):13-20. | Single cohort, no comparison |
| Wong J, Lee K-F, Yu SC-H, Lee PS-F, Cheung Y-S, Chong C-N, et al. Percutaneous radiofrequency ablation versus surgical radiofrequency ablation for malignant liver tumours: the long-term results. HPB. 2013;15(8):595-601. | No matching or multivariate analysis |
| Wong SL, Edwards MJ, Chao C, Simpson D, McMasters KM. Radiofrequency ablation for unresectable hepatic tumors. Am J Surg. 2001;182(6):552-7. | Single cohort, no comparison |
| Wong SL, Mangu PB, Choti MA, Crocenzi TS, Dodd GD, 3rd, Dorfman GS, et al. American Society of Clinical Oncology 2009 clinical evidence review on radiofrequency ablation of hepatic metastases from colorectal cancer. J Clin Oncol. 2010;28(3):493-508. | No quality appraisal of included studies |
| Wood TF, Rose DM, Chung M, Allegra DP, Foshag LJ, Bilchik AJ. Radiofrequency ablation of 231 unresectable hepatic tumors: indications, limitations, and complications. Ann Surg Oncol. 2000;7(8):593-600. | Wrong comparison |
| Wu Y-Z, Li B, Wang T, Wang S-J, Zhou Y-M. Radiofrequency ablation vs hepatic resection for solitary colorectal liver metastasis: a meta-analysis. World J Gastroenterol. 2011;17(36):4143-8. | No quality appraisal of included studies |
| Yang S, Alibhai SMH, Kennedy ED, El-Sedfy A, Dixon M, Coburn N, et al. Optimal management of colorectal liver metastases in older patients: a decision analysis. HPB. 2014;16(11):1031-42. | Markov model |
| Yokoyama T, Egami K, Miyamoto M, Watanabe H, Hasegawa H, Iida S, et al. Percutaneous and laparoscopic approaches of radiofrequency ablation treatment for liver cancer. Journal of Hepato Biliary Pancreatic Surgery. 2003;10(6):425-7. | Wrong comparison |
| Yoon HM, Kim JH, Shin YM, Won HJ, Kim PN. Percutaneous radiofrequency ablation using internally cooled wet electrodes for treatment of colorectal liver metastases. Clin Radiol. 2012;67(2):122-7. | Single cohort, no comparison |
| Zagoria RJ, Chen MYM, Shen P, Levine EA. Complications from radiofrequency ablation of liver metastases. Am Surg. 2002;68(2):204-9. | Single cohort, no comparison |
| Zdenkowski N, Chen S, Van Der westhuizen A, Ackland S. Curative strategies for liver metastases from colorectal cancer: A review. Oncologist. 2012;17(2):201-11. | Narrative review |
| Zhang K, Yu J, Zhou F, Yu X, Li X, et al. Impact of timing and cycles of systemic chemotherapy on survival outcome of colorectal liver metastases patients treated by percutaneous microwave ablation. International J of Hyperthermia. 2016;32(5):531-38 | Wrong comparison |
| Zhang X, Chen B, Hu S, Wang L, Wang K, Wachtel MS, et al. Microwave ablation with cooled-tip electrode for liver cancer: an analysis of 160 cases. Hepatogastroenterology. 2008;55(88):2184-7. | No separate results for CRLM |
